# Identification of the Molecular Mechanisms of Peimine in the Treatment of Cough Using Computational Target Fishing

**DOI:** 10.3390/molecules25051105

**Published:** 2020-03-02

**Authors:** Lihua Zhang, Mingchao Cui, Shaojun Chen

**Affiliations:** 1 Department of Food Science, Zhejiang Pharmaceutical College, Ningbo 315000, China; zhlihua111@126.com; 2 Department of Traditional Chinese Medicine, Zhejiang Pharmaceutical College, Ningbo 315000, China; cuimc@mail.zjpc.net.cn

**Keywords:** computational target fishing, cough, *Fritillariae Thunbergii Bulbus*, peimine, traditional Chinese medicine, verticine

## Abstract

Peimine (also known as verticine) is the major bioactive and characterized compound of *Fritillariae Thunbergii Bulbus*, a traditional Chinese medicine that is most frequently used to relieve a cough. Nevertheless, its molecular targets and mechanisms of action for cough are still not clear. In the present study, potential targets of peimine for cough were identified using computational target fishing combined with manual database mining. In addition, protein-protein interaction (PPI), gene ontology (GO) and Kyoto Encyclopedia of Genes and Genomes (KEGG) pathway enrichment analyses were performed using, GeneMANIA and Database for Annotation, Visualization and Integrated Discovery (DAVID) databases respectively. Finally, an interaction network of drug-targets-pathways was constructed using Cytoscape. The results identified 23 potential targets of peimine associated with cough, and suggested that MAPK1, AKT1 and PPKCB may be important targets of pemine for the treatment of cough. The functional annotations of protein targets were related to the regulation of immunological and neurological function through specific biological processes and related pathways. A visual representation of the multiple targets and pathways that form a network underlying the systematic actions of peimine was generated. In summary, peimine is predicted to exert its systemic pharmacological effects on cough by targeting a network composed of multiple proteins and pathways.

## 1. Introduction

Cough is a vital defense mechanism, especially for maintaining airway patency and to the elimination of potentially harmful stimuli from the airway and lung [1,2,3]. It can be acute, severe and life-threatening or, generally in most cases, troublesome and persistent, negative impact on daily activities and reducing life quality [1]. Problematic cough becomes to be one of the most common complaints for seeking medical attention [1,3,4]. For example, the common cold and influenza, probably the most common human disease, can result in a cough, which is one of its most irritating and persistent symptom, outlasting other symptoms in 69% of respondents in an internet survey [5,6]. Generally, acute cough is mostly caused by the common cold [4]. Subacute cough likely refers to a prolonged post-viral response [4]. Furthermore, chronic cough is one symptom of many kinds of diseases, including asthma/eosinophilic bronchitis, rhinitis, gastro-oesophageal acid reflux disease, and so on [7]. Its underlying mechanisms can be understood as linked to different patterns of lower airway inflammation and to laryngeal hypersensitivity [8]. Additionally, cough treatments lead to huge financial costs and health-care resources consumption [4].

On account of multifactorial causes, multifaceted cough therapies, pharmacologic and nonpharmacologic, includes avoidance of allergens/environmental triggers/irritants, dietary and lifestyle modifications, behavioral exercises, nasal sprays, airway clearance, respiratory inhales, and drugs treatment [5,9]. Currently, many new potential compounds, especially acting on nervous system, such as voltage-gated sodium channels blocker (NaV), TRP channel blockers (TRPVs), neuromodulators, purinergic antagonists (P2 × 2/P2X3), neurokinin antagonists (Substance P), GABAB receptor agonists, a7 acetylcholine receptor agonists, and so on, are under developing [10,11]. To date, effective anti-tussive agents are still desired and becoming an unmet clinical need [4].

**Figure 1:**
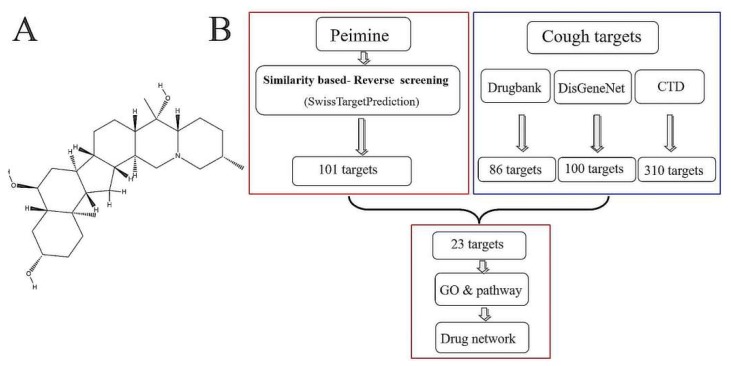
(**A**) Chemical structure of Peimine from the PubChem database (CID: 131900). (**B**) Workflow of the identification of putative peimine targets that integrates target fishing, databases mining, gene ontology (GO) analyses, pathway analyses and network construction.

As a complementary and alternative medicine, *Fritillariae Thunbergii Bulbus* (FTB), listed in the Pharmacopeia of the People’s Republic of China, has been widely used to suppress cough and resolve phlegm for thousands of years [12,13]. The alkaloid peimine (also called verticine) (Figure 1A), is the major bioactive and characterized compound of FTB [13,14]. Our previous study used gas chromatography-mass spectrometry to show that peimine is abundant in the bulbs, flowers, leaves, and stems of FTB [15]. Peimine has several pharmacological benefits, including anticancer and anti-inflammatory actions, protection against acute lung injury, and promotion of tracheobronchial relaxation as well as antitussive, expectorant, sedative, and analgesic activities [14]. However, most of the therapeutic targets and mechanisms of actions of peimine in relief from cough remain unclear, which impedes the discovery of novel drugs from natural products.

Target identification of chemical bioactive compounds is at the heart of drug discovery and development [16]. The information of binding targets will help to understand the mechanisms of drug action, improve the clinical application, explain off-target effects, and allow for better analog design [17]. Compared to the traditional experimental approaches, which are labor-, resource- and time-intensive, computer-aided drug discovery becomes more efficient and economic, being an attractive complementary method in drug development [18,19]. Therefore, computational approaches have the potential to assist in the unraveling of the mechanisms of peimine-mediated cough relief.

In the present study, we investigated the potential targets and mechanisms of actions of peimine for the treatment of cough using computational target fishing (Figure 1B). First, the potential targets of peimine were predicted using the SwissTargetPrediction database. Then, we collected the potential targets for cough from three different databases. The targets identified that were common to both approaches were selected for further investigation using protein-protein interactions (PPIs), gene ontology (GO), and pathway-enrichment analysis. A drug-target network was constructed to provide a systematic overview of the potential targets of peimine. These results may clarify why peimine is effective in the relief of cough and may facilitate the development of novel drugs for this indication.

## 2. Results

### 2.1. Potential Targets of Peimine Identified by SwissTargetPrediction

**Figure 2:**
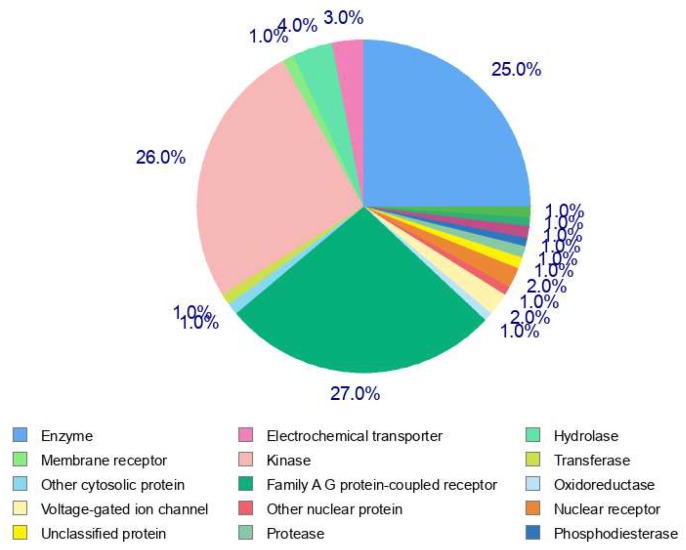
Pie chart of 101 predicted targets class.

Sorted by the probability values, one hundred potential protein targets of peimine were identified using SwissTargetPrediction. Due to the diversity of gene classification, farnesyltransferase subtypes (FNTA and FNTB) were combined into one result, resulting in addition of one additional target to account for the two subtypes of this enzyme. Therefore, we identified 101 potential targets for peimine (Supplementary File, S1). Of the predicted targets classes, 27% of targets belonged to family A G protein-coupled receptor, 26% to kinases, and 25% to enzymes (Figure 2). Table 1 lists the top 15 putative targets of peimine, showing that the targets lanosterol synthase, vesicular acetylcholine transporter (SLC18A3) and beta-glucocerebrosidase ranked as the top three targets, respectively.

### 2.2. Method Validation

The Drugbank database showed that compound R048-8071 is the original ligand of lanosterol synthase. We copied the R048-8071 structure file to the SwissTargetPrediction server to identify its potential targets, and as expected lanosterol synthase was the top predicted target of R048-8071 (Table 2). The targets for the five approved anti-cough drugs, chlorpheniramine, alimemazine, dextromethorphan, homatropine, montelukase and the classic drug aspirin were also as expected (Table 2; raw data are listed Supplementary File S2), indicating that the SwissTargetPrediction database is suitable for target identification.

### 2.3. Screening for the Target Proteins of Peimine for Cough

**Figure 3:**
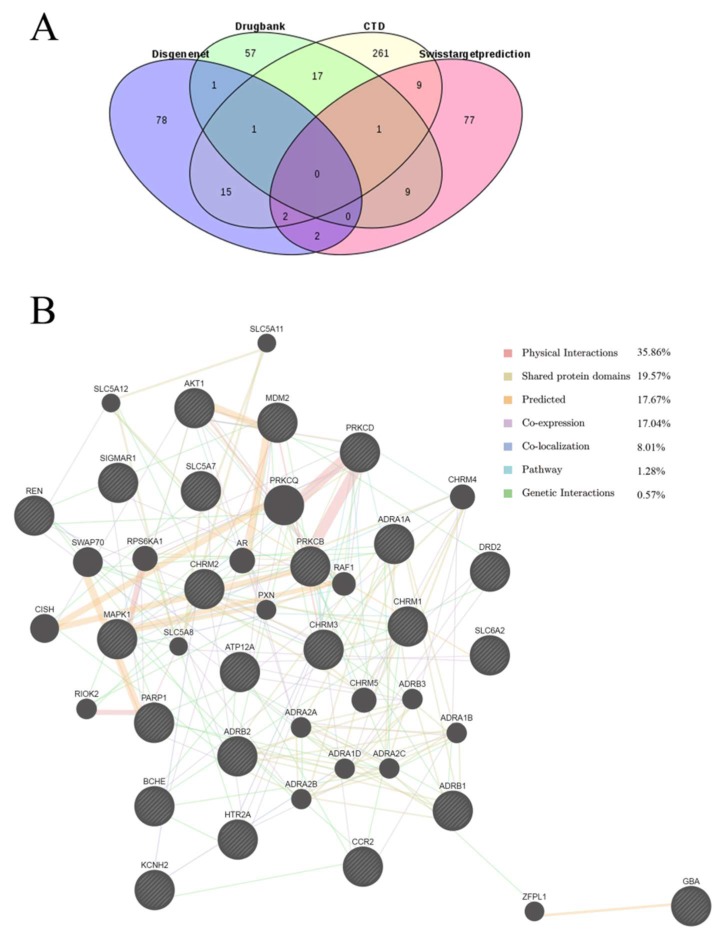
The 23 targets of peimine for the treatment of cough. (**A**) Intersected targets using Venn. (**B**) Protein network analysis using GeneMANIA.

Potential protein targets related to cough were identified from the Drugbank, Comparative Toxicogenomics Database (CTD), and DisGeNET databases using the search terms ‘cough’ and/or ‘productive cough’ and ‘Homo sapiens’. Eighty-six targets were obtained from Drugbank, 310 from CTD database, and 100 targets from DisGeNET database (Supplementary File S3). After manual checking and deletion of duplicates, a total of 458 cough-related targets remained. Venn diagram analysis, in which 101 predicted protein targets of peimine were overlaid on 458 cough-related targets, identified 23 intersected proteins (Figure 3A and Table 3). Of these 23 proteins and the proteins that they interact with in the PPI network, 35.86% are engaged in physical interactions with their interacting partners and 19.57% share a protein domain with their interacting partners (Figure 3B). Taken together, these results suggested that that these targets and their interacting proteins may have identical or similar functions.

### 2.4. GO Analysis and Network Construction

**Figure 4:**
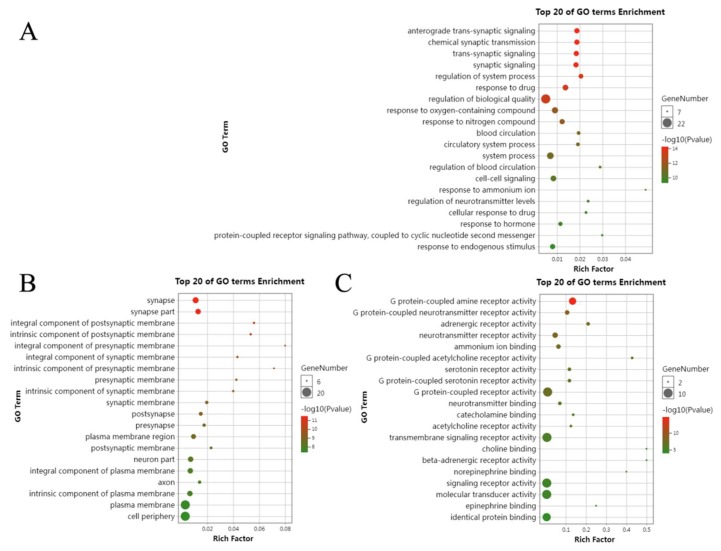
Gene ontology (GO) map of 23 targets of peimine for the treatment of cough. (**A**) Biological process (BP), (**B**) cellular component (CC), (**C**) molecular function (MF).

The GO uses a common controlled vocabulary to annotate and unify the homologous gene and proteins in multiple species, making it possibly and easily to query and retrieve shared genes and proteins, becoming very useful tools in computational biological research [20]. It contains three categories: biological process (BP), molecular function (MF) and cellular component (CC) [20]. GO enrichment analysis showed that the 23 targets of peimine are involved in biological processes such as anterograde trans-synaptic signaling, chemical synaptic transmission, trans-synaptic signaling, synaptic signaling and regulation of system processes (Figure 4A). Molecular functions of these proteins include G protein-coupled amine receptor activity, G protein-coupled neurotransmitter receptor activity, adrenergic receptor activity, neurotransmitter receptor activity, and ammonium ion binding (Figure 4C).

**Figure 5:**
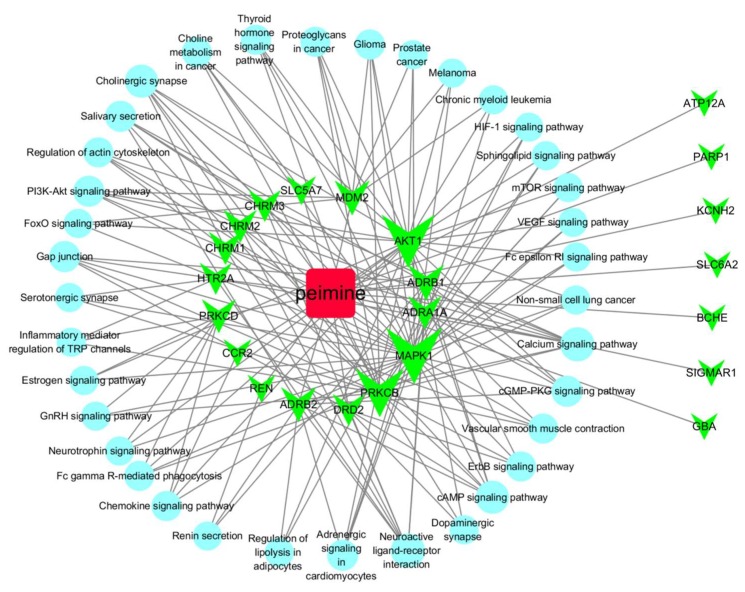
Drug-target-pathway network of peimine for the treatment of cough. Red oblong: peimine, green triangles: target proteins, cyan circles: pathways. The size of node is related to node degree.

For interaction network of gene products, Kyoto Encyclopedia of Genes and Genomes (KEGG) pathway provides systematic analysis and reveals the higher order biological information of gene functions [21]. These targets were also enriched in 37 KEGG pathways (*p* < 0.05), including calcium signaling pathway, cholinergic synapse, neuroactive ligand-receptor interaction, cAMP signaling pathway, and salivary secretion (Supplementary File S4). Based on target fishing and pathway analysis, an entire network was constructed using Cytoscape. As shown in Figure 5, the interaction network has 60 nodes and 168 edges. Topological analysis of this network showed that MAPK1, AKT1 and PPKCB have higher degrees in the network. Therefore, MAPK1, AKT1 and PPKCB may have pivotal roles in the mechanism of action of pemine in the treatment of cough.

## 3. Discussion

Herbal medicine and traditional Chinese medicine (TCM) have been used to fight against many kinds of diseases from the ancient times to today [18]. Nevertheless, most of the pharmacological targets for bioactive natural compounds s are still unclear, which greatly delays the application and development of TCM [18]. Various computational methods are available to complement experimental ‘wet-lab’ approaches in the identification of drug targets [19,22]. Here, we found 23 targets of peimine in cough relief using computational target fishing combined with manual database mining (Table 3). Most of the proteins we identified are related to various neuropeptide receptors, signal peptides and receptors for inflammatory mediators (Table 3). Our results complement ongoing research into novel neuroimmune-targeted antitussive therapies [3,10].

MAPKs, AKT and PPKCB may be targets of peimine for cough relief that act as hub proteins in the network (Figure 5). MAP kinases participate signals transduction in many cellular pathways and respond to different kinds of cellular stress [23]. Peimine inhibits IL-1β-induced inflammation in mouse chondrocytes through suppressing the MAPK pathway activation, and plays pharmacological effects in a mouse model of osteoarthritis [24]. In addition, peimine inhibits the production of inflammatory cytokines induced by lipopolysaccharide by blocking MAPK and NF-kB signaling pathways [25]. Another major biologically active alkaloid in FTB is peiminine, which shares the same pharmacophore as peimine [13]. This compound acts on pathways involving Akt signaling. Peiminine induces cell cycle arrest through inhibiting Akt-GSk3β signaling pathway, and decreases autophagic flux via depressing AMPK-ULK1 signaling pathway in glioblastoma multiforme cells [26]. It also inhibits the proliferation of colorectal cancer cells via activation of metabolic pathways related to the regulation of the PI3K-Akt-mTOR pathway and oxidative stress [27]. In addition, peiminine protects against LPS-induced mastitis by inhibiting the Akt-NF-κB, ERK1/2 and p38 signaling pathways; therefore, it might be a potential therapeutic agent for mastitis [23]. These findings suggest peimine may exert pharmacological effects through multiple targets rather than a single protein.

The mechanisms of acute or chronic cough are complex and involve heterogeneous mechanisms, including lower airway inflammation (bronchitis), non-eosinophilic asthma and sensory hyperresponsiveness [3,7,8]. Results presented in Figure 4 suggest that peimine may work through various pathways, such as the chemokine signaling pathway, the Fc epsilon RI signaling pathway, and inflammatory mediator regulation of transient receptor potential channels. All of these pathways are closely related to inflammatory actions. Many other pathways are related to neural actions, such as cholinergic synapse, adrenergic signaling in cardiomyocytes, serotonergic synapse, neurotropin signaling pathway, and dopaminergic synapse (Figure 5). Therefore, peimine may relieve cough through suppression of inflammatory or neural pathways, which suggests that the underlying mechanisms of cough are linked to different patterns of lower airway inflammation and to laryngeal hypersensitivity [10]. In addition, the calcium signaling pathway was the top pathway in our results (Supplementary File S4 and Figure 5), which is consistent with findings showing that calcium signaling pathways are involved in cough and the actions of peimine. Calcium influx or activation of calcium-activated potassium channels may contribute to cough reflex sensitivity induced by IFN-γ or capsaicin [28,29]. In prostate cancer cells, peimine can inhibit cell growth and motility, and induce apoptosis by disruption of intracellular calcium homeostasis through the Ca^2+^/CaMKII/JNK pathway [30]. Therefore, peimine may exert effects through multiple targets and multiple pathways for cough relief. In addition, owning to multifactorial causes of cough, and the polypharmacology of natural compounds, it is hard to clarify one certain target or pathway. Therefore, we constructed the ‘drug-target-pathway’ network to give a quick visual view of action mechanisms of peimine for cough treatment (Figure 5).

Based on the hypothesis ‘common proteins targets by similar molecules’ and measures of a combination of 2D and 3D similarity with known ligand, SwissTargetPrediction show high performance in target fishing for bioactive small molecule [31,32,33]. With their known protein targets, the five approved anti-cough drugs and the classic drug aspirin were used to method validation. The anticipated results indicated that the method have very good accuracy (Table 2). Therefore, it may be a powerful tool in this research. However, it doesn’t go well for the inactive compounds that exhibit good similarity with active compounds, or to detect subtle differences between molecules [32,33]. Therefore, target predictions for other compounds should be carefully interpreted.

In conclusion, the putative targets of peimine were identified by computational target fishing and manual database mining. Our results showed that peimine may act on 23 protein targets that are associated with its therapeutic suppression of cough. In addition, GO and pathway enrichment analysis were preformed and a drug-target-pathway network was constructed. These results indicate that peimine may exert pharmacological effects on a systemic network of proteins and pathways to relieve cough. Although further studies are needed to support our findings, this study provides a systemic and visual view of the possible molecular mechanisms and signaling pathways that contribute to the mechanisms of peimine in the treatment of cough.

## 4. Materials and Methods

### 4.1. Target Identification of Peimine by Computational Target Fishing

Computational approaches have the significant advantages to greatly reduce times and costs required for drug target fishing [17]. SwissTargetPrediction (http://www.swisstargetprediction.ch/) can predict the most probable protein targets for bioactive small molecules based on the similarity hypothesis (i.e., that two similar molecules are likely to have the same protein target) through reverse virtual screening [32,33]. Using 2D and 3D similarity combined measures, the target predictions can fish similar molecules within a data set possessing 376,342 compounds that are experimentally proved to active on another set of 3068 protein targets [32,33]. The canonical SMILES of peimine (PubChem CID: 131900) was copied from the PubChem database and uploaded to the SwissTargetPrediction server. For one approved drug, its known targets can be compared with the predicted. Therefore, five approved anti-cough drugs (chlorpheniramine, alimemazin, dextromethorphan, homatropine and montelukase), aspirin, and the original ligand predicted to be the top target of peimine were used as controls to validate the methods.

### 4.2. Collection of Target Proteins Associated with Cough

Protein targets related to cough were obtained through searches on the Drugbank database (https://www.drugbank.ca/), the Comparative Toxicogenomics Database (CTD, http://ctdbase.org/) and the DisGeNET database (https://www.disgenet.org/) using the keywords “cough” or “phlegm (also named productive cough)”. All the cough targets identified in the DisGeNET and Drugbank database were retained after manual mining because the number of potential targets was not high (no more than 100 targets from each database). However, because the number of cough targets identified in the CTD database was very large, we chose targets that had an inference score >30.0 (a higher inference score indicates a greater correlation between the active compound and the target) [34].

### 4.3. Protein Targets of Peimine Associated with Cough

PPls plays a crucial role in the regulation of biological systems, and certain class of PPI are amenable to small-molecule inhibition [35]. Proteins that were common to both the result sets of computational target fishing and cough targets identified by manual database searching (referred to as intersected proteins), that is, targets of peimine associated with cough, were identified using Venn diagram software in OmicShare tools (https://www.omicshare.com/tools/). GeneMAINA (http://genemania.org/) were used to predict potential PPI networks from these intersected proteins, and the selection parameter for species was set to “Homo sapiens”.

### 4.4. GO Analysis and Network Construction

To investigate the meaningful biological functional annotation of these potential targets, GO enrichment analysis was used to extract the key GO terms (BP, MF, and CC) on the OmicShare platform, and KEGG pathways based on the Database for Annotation, Visualization and Integrated Discovery (DAVID) (https://david.ncifcrf.gov/). Pathways that had significant changes of *p* < 0.05 were chosen for construction of the drug-target-pathway network.

## Figures and Tables

**Table 1:** The top 15 putative targets of peimine identified by SwissTargetPrediction.

Rank	Target	Common Name	Uniprot ID
1	Lanosterol synthase	LSS	P48449
2	Vesicular acetylcholine transporter	SLC18A3	Q16572
3	Beta-glucocerebrosidase	GBA	P04062
4	Lysosomal alpha-glucosidase (by homology)	GAA	P10253
5	Phospholipase A2 group 1B	PLA2G1B	P04054
6	Sigma opioid receptor	SIGMAR1	Q99720
7	Neutral alpha-glucosidase AB	GANAB	Q14697
8	Alpha-L-fucosidase I	FUCA1	P04066
9	Estradiol 17-beta-dehydrogenase 3	HSD17B3	P37058
10	Anti-estrogen binding site (AEBS)	EBP	Q15125
11	Alpha-L-fucosidase 2	FUCA2	Q9BTY2
12	Neutral alpha-glucosidase C	GANC	Q8TET4
13	Alpha-galactosidase A	GLA	P06280
14	Beta-glucosidase	GBA2	Q9HCG7
15	Serine/threonine-protein kinase AKT2	AKT2	P31751

**Table 2:** The SwissTargetPrediction results of control compounds for method validation.

Drug	Original Target	Rank in Prediction Results
Aspirin	Cyclooxygenase-1, PTGS1	1
Chlorpheniramine	Histamine H1 receptor, HRH1	1
Alimemazine	Histamine H1 receptor, HRH1	13
Dextromethorphan	Sigma non-opioid intracellular receptor 1, SIGMAR1	2
Homatropine	Muscarinic acetylcholine receptor M1, CHRM1	4
montelukast	Cysteinyl leukotriene receptor 1, CYSLTR1	2
R048-8071	Lanosterol synthase, LSS	1

**Table 3:** Potential 23 targets of peimine related to cough.

Rank	Target	Common Name	Uniprot ID
1	Beta-glucocerebrosidase	GBA	P04062
2	Sigma opioid receptor	SIGMAR1	Q99720
3	Serine/threonine-protein kinase AKT	AKT1	P31749
4	Butyrylcholinesterase	BCHE	P06276
5	Dopamine D2 receptor	DRD2	P14416
6	Adrenergic receptor beta	ADRB2	P07550
7	Muscarinic acetylcholine receptor M1	CHRM1	P11229
8	Muscarinic acetylcholine receptor M3	CHRM3	P20309
9	Alpha-1a adrenergic receptor	ADRA1A	P35348
10	Protein kinase C beta	PRKCB	P05771
11	p53-binding protein Mdm-2	MDM2	Q00987
12	Serotonin 2a (5-HT2a) receptor	HTR2A	P28223
13	Norepinephrine transporter	SLC6A2	P23975
14	Muscarinic acetylcholine receptor M2	CHRM2	P08172
15	HERG	KCNH2	Q12809
16	Protein kinase C delta	PRKCD	Q05655
17	MAP kinase ERK2	MAPK1	P28482
18	C-C chemokine receptor type 2	CCR2	P41597
19	Beta-1 adrenergic receptor	ADRB1	P08588
20	Poly [ADP-ribose] polymerase-1	PARP1	P09874
21	High-affinity choline transporter (by homology)	SLC5A7	Q9GZV3
22	Renin	REN	P00797
23	Potassium-transporting ATPase alpha chain 2	ATP12A	P54707

## Supplementary Materials

The following are available online https://www.mdpi.com/1420-3049/25/5/1105/s1, Supplementary File S1: the SwissTargetPrediction result of peimine; Supplementary File S2: the SwissTargetPrediction results of four control compounds; Supplementary File S3: the cough-related targets collected from public databases; Supplementary File S4: the KEGG pathways for 23 targets of peimine for the treatment of cough.
