# Supplementary material for: Identification of the Molecular Mechanisms of Peimine in the Treatment of Cough Using Computational Target Fishing
Source: Molecules. 2020 Mar 2;25(5):1105. doi: 10.3390/molecules25051105 (PMC7179178; doi:10.3390/molecules25051105)
Supplement: Supplementary file 1 [file molecules-25-01105-s001.zip › SwissTargetPrediction results of Alimemazine.pdf]

SwissTargetPrediction

Target	Common name	Uniprot ID	ChEMBL ID	Target Class	Probability*	Known actives (3D/2D)
Muscarinic acetylcholine receptor M4	CHRM4	P08173	CHEMBL1821	Family A G protein-coupled receptor	0.999621429154	30 / 9
Serotonin 2b (5-HT2b) receptor	HTR2B	P41595	CHEMBL1833	Family A G protein-coupled receptor	0.999621429154	82 / 12
Alpha-2a adrenergic receptor	ADRA2A	P08913	CHEMBL1867	Family A G protein-coupled receptor	0.999621429154	27 / 9
Alpha-2b adrenergic receptor	ADRA2B	P18089	CHEMBL1942	Family A G protein-coupled receptor	0.999621429154	22 / 9
Muscarinic acetylcholine receptor M5	CHRM5	P08912	CHEMBL2035	Family A G protein-coupled receptor	0.999621429154	32 / 9
Muscarinic acetylcholine receptor M2	CHRM2	P08172	CHEMBL211	Family A G protein-coupled receptor	0.999621429154	43 / 9
Muscarinic acetylcholine receptor M1	CHRM1	P11229	CHEMBL216	Family A G protein-coupled receptor	0.999621429154	74 / 9
Norepinephrine transporter	SLC6A2	P23975	CHEMBL222	Electrochemical transporter	0.999621429154	342 / 10
Alpha-1d adrenergic receptor	ADRA1D	P25100	CHEMBL223	Family A G protein-coupled receptor	0.999621429154	15 / 9
Serotonin 2a (5-HT2a) receptor	HTR2A	P28223	CHEMBL224	Family A G protein-coupled receptor	0.999621429154	304 / 17
Serotonin 2c (5-HT2c) receptor	HTR2C	P28335	CHEMBL225	Family A G protein-coupled receptor	0.999621429154	229 / 15
Alpha-1a adrenergic receptor (by homology)	ADRA1A	P35348	CHEMBL229	Family A G protein-coupled receptor	0.999621429154	31 / 9
Histamine H1 receptor	HRH1	P35367	CHEMBL231	Family A G protein-coupled receptor	0.999621429154	59 / 12
Dopamine D3 receptor	DRD3	P35462	CHEMBL234	Family A G protein-coupled receptor	0.999621429154	125 / 16
Muscarinic acetylcholine receptor M3	CHRM3	P20309	CHEMBL245	Family A G protein-coupled receptor	0.999621429154	35 / 9
Sigma opioid receptor	SIGMAR1	Q99720	CHEMBL287	Membrane receptor	0.999621429154	203 / 14
Serotonin 6 (5-HT6) receptor	HTR6	P50406	CHEMBL3371	Family A G protein-coupled receptor	0.999621429154	399 / 20
Adrenergic receptor alpha-2	ADRA2C	P18825	CHEMBL1916	Family A G protein-coupled receptor	0.906314705411	20 / 9

Target	Common name	Uniprot ID	ChEMBL ID	Target Class	Probability*	Known actives (3D/2D)
Histamine H2 receptor	HRH2	P25021	CHEMBL1941	Family A G protein-coupled receptor	0.906314705411	13 / 9
Dopamine D1 receptor	DRD1	P21728	CHEMBL2056	Family A G protein-coupled receptor	0.906314705411	31 / 11
Dopamine D2 receptor	DRD2	P14416	CHEMBL217	Family A G protein-coupled receptor	0.906314705411	251 / 18
Serotonin transporter	SLC6A4	P31645	CHEMBL228	Electrochemical transporter	0.906314705411	532 / 45
Cytochrome P450 2D6	CYP2D6	P10635	CHEMBL289	Cytochrome P450	0.906314705411	6 / 4
Prion protein	PRNP	P04156	CHEMBL4869	Surface antigen	0.906314705411	3 / 4
Mucosa-associated lymphoid tissue lymphoma translocation protein 1	MALT1	Q9UDY8	CHEMBL3632452	Hydrolase	0.361228277245	1 / 1
Tyrosine-protein kinase FYN	FYN	P06241	CHEMBL1841	Kinase	0.221330189598	3 / 4
Dopamine D5 receptor	DRD5	P21918	CHEMBL1850	Family A G protein-coupled receptor	0.221330189598	19 / 3
Serotonin 1b (5-HT1b) receptor (by homology)	HTR1B	P28222	CHEMBL1898	Family A G protein-coupled receptor	0.221330189598	83 / 6
Serotonin 1a (5-HT1a) receptor	HTR1A	P08908	CHEMBL214	Family A G protein-coupled receptor	0.221330189598	123 / 42
Dopamine D4 receptor	DRD4	P21917	CHEMBL219	Family A G protein-coupled receptor	0.221330189598	48 / 4
Potassium channel subfamily K member 2	KCNK2	O95069	CHEMBL2321615	Voltage-gated ion channel	0.221330189598	1 / 2
Neurokinin 2 receptor	TACR2	P21452	CHEMBL2327	Family A G protein-coupled receptor	0.221330189598	1 / 2
Mu opioid receptor	OPRM1	P35372	CHEMBL233	Family A G protein-coupled receptor	0.221330189598	50 / 2
Delta opioid receptor	OPRD1	P41143	CHEMBL236	Family A G protein-coupled receptor	0.221330189598	16 / 2
Kappa Opioid receptor	OPRK1	P41145	CHEMBL237	Family A G protein-coupled receptor	0.221330189598	29 / 3
Dopamine transporter	SLC6A3	Q01959	CHEMBL238	Electrochemical transporter	0.221330189598	396 / 20
HERG	KCNH2	Q12809	CHEMBL240	Voltage-gated ion channel	0.221330189598	70 / 7
Serotonin 7 (5-HT7) receptor	HTR7	P34969	CHEMBL3155	Family A G protein-coupled receptor	0.221330189598	73 / 6
P-glycoprotein 1	ABCB1	P08183	CHEMBL4302	Primary active	0.221330189598	1 / 2

Target	Common name	Uniprot ID	ChEMBL ID	Target Class	Probability*	Known actives (3D/2D)
				transporter		
Melanocortin receptor 5	MC5R	P33032	CHEMBL4608	Family A G protein-coupled receptor	0.221330189598	2 / 2
Calmodulin	CALM1	P62158	CHEMBL6093	Unclassified protein	0.221330189598	1 / 2
Vanilloid receptor (by homology)	TRPV1	Q8NER1	CHEMBL4794	Voltage-gated ion channel	0.221330189598	1 / 1
Serine/threonine-protein kinase PIM1	PIM1	P11309	CHEMBL2147	Kinase	0.196657136121	7 / 6
Butyrylcholinesterase	BCHE	P06276	CHEMBL1914	Hydrolase	0.188420385827	11 / 70
Acetylcholinesterase	ACHE	P22303	CHEMBL220	Hydrolase	0.188420385827	7 / 18
Cyclooxygenase-1	PTGS1	P23219	CHEMBL221	Oxidoreductase	0.114337558605	0 / 3
Cyclooxygenase-2	PTGS2	P35354	CHEMBL230	Oxidoreductase	0.114337558605	0 / 4
Cytochrome P450 1A2	CYP1A2	P05177	CHEMBL3356	Cytochrome P450	0.0978745343258	1 / 3
Histone-lysine N-methyltransferase SETD7	SETD7	Q8WTS6	CHEMBL5523	Writer	0.0978745343258	3 / 0
Monoamine oxidase A	MAOA	P21397	CHEMBL1951	Oxidoreductase	0.0978745343258	31 / 1
Epidermal growth factor receptor erbB1	EGFR	P00533	CHEMBL203	Kinase	0.0978745343258	2 / 1
Cytochrome P450 2C19	CYP2C19	P33261	CHEMBL3622	Cytochrome P450	0.0978745343258	3 / 1
Neuronal acetylcholine receptor protein alpha-7 subunit	CHRNA7	P36544	CHEMBL2492	Ligand-gated ion channel	0.0978745343258	6 / 0
Anti-estrogen binding site (AEBS)	EBP	Q15125	CHEMBL4931	Enzyme	0.0978745343258	1 / 1
Adenosine A3 receptor	ADORA3	P0DMS8	CHEMBL256	Family A G protein-coupled receptor	0.0978745343258	2 / 1
Serotonin 3a (5-HT3a) receptor	HTR3A	P46098	CHEMBL1899	Ligand-gated ion channel	0.0978745343258	24 / 36
Serotonin 1e (5-HT1e) receptor	HTR1E	P28566	CHEMBL2182	Family A G protein-coupled receptor	0.0978745343258	7 / 0
Alpha-1b adrenergic receptor	ADRA1B	P35368	CHEMBL232	Family A G protein-coupled receptor	0.0978745343258	7 / 0
Sodium channel protein type V alpha subunit	SCN5A	Q14524	CHEMBL1980	Voltage-gated ion channel	0.0978745343258	1 / 0
Kinesin-1 heavy chain/ Tyrosine-protein kinase receptor RET	RET	P07949	CHEMBL2041	Kinase	0.0978745343258	4 / 0
Sodium channel protein type II alpha subunit	SCN2A	Q99250	CHEMBL4187	Voltage-gated ion channel	0.0978745343258	2 / 0
Sodium channel protein type IX alpha subunit	SCN9A	Q15858	CHEMBL4296	Voltage-gated ion channel	0.0978745343258	1 / 0
Vitamin D receptor	VDR	P11473	CHEMBL1977	Nuclear receptor	0.0978745343258	3 / 0
Monoamine oxidase B	MAOB	P27338	CHEMBL2039	Oxidoreductase	0.0978745343258	22 / 0

Target	Common name	Uniprot ID	ChEMBL ID	Target Class	Probability*	Known actives (3D/2D)
Interferon-alpha/beta receptor alpha chain	IFNAR1	P17181	CHEMBL1887	Membrane receptor	0.0978745343258	1 / 0
Nociceptin receptor	OPRL1	P41146	CHEMBL2014	Family A G protein-coupled receptor	0.0978745343258	37 / 0
C-X-C chemokine receptor type 3	CXCR3	P49682	CHEMBL4441	Family A G protein-coupled receptor	0.0978745343258	7 / 0
Emopamil-binding protein-like	EBPL	Q9BY08	CHEMBL2311238	Unclassified protein	0.0978745343258	0 / 1
LSD1/CoREST complex	RCOR1 KDM1A	Q9UKL0 O60341	CHEMBL3137262	Eraser	0.0978745343258	17 / 0
Beta-3 adrenergic receptor	ADRB3	P13945	CHEMBL246	Family A G protein-coupled receptor	0.0978745343258	0 / 2
Neurokinin 1 receptor (by homology)	TACR1	P25103	CHEMBL249	Family A G protein-coupled receptor	0.0978745343258	4 / 0
Kinesin-like protein 1	KIF11	P52732	CHEMBL4581	Other cytosolic protein	0.0978745343258	27 / 3
Cytochrome P450 3A4	CYP3A4	P08684	CHEMBL340	Cytochrome P450	0.0978745343258	1 / 0
Progesterone receptor	PGR	P06401	CHEMBL208	Nuclear receptor	0.0978745343258	10 / 0
Sodium channel protein type IV alpha subunit	SCN4A	P35499	CHEMBL2072	Voltage-gated ion channel	0.0978745343258	4 / 0
Protein farnesyltransferase	FNTA FNTB	P49354 P49356	CHEMBL2094108	Enzyme	0.0978745343258	0 / 3
Choline acetylase	CHAT	P28329	CHEMBL4039	Enzyme	0.0978745343258	1 / 0
Serotonin 1f (5-HT1f) receptor	HTR1F	P30939	CHEMBL1805	Family A G protein-coupled receptor	0.0978745343258	9 / 0
Intermediate conductance calcium-activated potassium channel protein 4	KCNN4	O15554	CHEMBL4305	Voltage-gated ion channel	0.0978745343258	2 / 0
Histamine H3 receptor	HRH3	Q9Y5N1	CHEMBL264	Family A G protein-coupled receptor	0.0	39 / 5
Androgen Receptor (by homology)	AR	P10275	CHEMBL1871	Nuclear receptor	0.0	0 / 4
Neuronal acetylcholine receptor; alpha4/beta2	CHRNA4 CHRNB2	P43681 P17787	CHEMBL1907589	Ligand-gated ion channel	0.0	22 / 0
Dipeptidyl peptidase IV	DPP4	P27487	CHEMBL284	Protease	0.0	19 / 0
Lysine-specific histone demethylase 1	KDM1A	O60341	CHEMBL6136	Eraser	0.0	20 / 0
Estrogen receptor beta	ESR2	Q92731	CHEMBL242	Nuclear receptor	0.0	1 / 0
Protein tyrosine kinase 2 beta	PTK2B	Q14289	CHEMBL5469	Kinase	0.0	1 / 0
UDP-glucuronosyltransferase 2B7	UGT2B7	P16662	CHEMBL4370	Enzyme	0.0	1 / 0

Target	Common name	Uniprot ID	ChEMBL ID	Target Class	Probability*	Known actives (3D/2D)
Lethal(3)malignant brain tumor-like protein 3	L3MBTL3	Q96JM7	CHEMBL1287623	Reader	0.0	4 / 0
S-100 protein beta chain	S100B	P04271	CHEMBL4300	Other cytosolic protein	0.0	2 / 0
Glutamate [NMDA] receptor subunit epsilon 2	GRIN2B	Q13224	CHEMBL1904	Ligand-gated ion channel	0.0	3 / 0
Lysosomal Pro-X carboxypeptidase	PRCP	P42785	CHEMBL2335	Protease	0.0	4 / 0
Nitric oxide synthase, inducible	NOS2	P35228	CHEMBL4481	Enzyme	0.0	4 / 0
Serotonin 1d (5-HT1d) receptor	HTR1D	P28221	CHEMBL1983	Family A G protein-coupled receptor	0.0	84 / 1
Cytochrome b-245 heavy chain	CYBB	P04839	CHEMBL1287627	Transmembrane 1-electron transfer carriers	0.0	3 / 0
Neuronal acetylcholine receptor; alpha4/beta4	CHRNB4 CHRNA4	P30926 P43681	CHEMBL1907591	Ligand-gated ion channel	0.0	1 / 0
Neuronal acetylcholine receptor; alpha3/beta4	CHRNA3 CHRNB4	P32297 P30926	CHEMBL1907594	Ligand-gated ion channel	0.0	8 / 0
Neuronal acetylcholine receptor; alpha3/beta2	CHRNA3 CHRNB2	P32297 P17787	CHEMBL2109234	Ligand-gated ion channel	0.0	2 / 0
Nicotinic acetylcholine receptor alpha2/beta4	CHRNB4 CHRNA1	P30926 P02708	CHEMBL3038459	Ligand-gated ion channel	0.0	1 / 0
Myeloperoxidase	MPO	P05164	CHEMBL2439	Enzyme	0.0	7 / 0
MAP kinase p38 alpha	MAPK14	Q16539	CHEMBL260	Kinase	0.0	1 / 0
