# Supplementary material for: Identification of the Molecular Mechanisms of Peimine in the Treatment of Cough Using Computational Target Fishing
Source: Molecules. 2020 Mar 2;25(5):1105. doi: 10.3390/molecules25051105 (PMC7179178; doi:10.3390/molecules25051105)
Supplement: Supplementary file 1 [file molecules-25-01105-s001.zip › SwissTargetPrediction Results of aspirin.pdf]

SwissTargetPrediction

Target	Common name	Uniprot ID	ChEMBL ID	Target Class	Probability*	Known actives (3D/2D)
Cyclooxygenase-1	PTGS1	P23219	CHEMBL221	Oxidoreductase	1.0	3 / 2
Cyclooxygenase-2	PTGS2	P35354	CHEMBL230	Oxidoreductase	1.0	5 / 4
Dopamine transporter	SLC6A3	Q01959	CHEMBL238	Electrochemical transporter	0.124537699969	0 / 2
Carbonic anhydrase II	CA2	P00918	CHEMBL205	Lyase	0.080569968052	29 / 38
Carbonic anhydrase I	CA1	P00915	CHEMBL261	Lyase	0.080569968052	32 / 37
Lysine-specific demethylase 4C	KDM4C	Q9H3R0	CHEMBL6175	Eraser	0.080569968052	20 / 0
Carbonic anhydrase VII	CA7	P43166	CHEMBL2326	Lyase	0.0717871625222	9 / 26
Carbonic anhydrase XII	CA12	O43570	CHEMBL3242	Lyase	0.0717871625222	23 / 30
Carbonic anhydrase XIV	CA14	Q9ULX7	CHEMBL3510	Lyase	0.0717871625222	10 / 26
Carbonic anhydrase IX	CA9	Q16790	CHEMBL3594	Lyase	0.0717871625222	22 / 28
Lysine-specific demethylase 4D-like	KDM4E	B2RXH2	CHEMBL1293226	Eraser	0.0717871625222	9 / 0
Lysine-specific demethylase 5C	KDM5C	P41229	CHEMBL2163176	Eraser	0.0717871625222	9 / 0
Lysine-specific demethylase 4A	KDM4A	O75164	CHEMBL5896	Eraser	0.0717871625222	11 / 0
Lysine-specific demethylase 4D	KDM4D	Q6B0I6	CHEMBL6138	Eraser	0.0717871625222	6 / 0
Hydroxycarboxylic acid receptor 2	HCAR2	Q8TDS4	CHEMBL3785	Family A G protein-coupled receptor	0.0717871625222	28 / 0
HM74 nicotinic acid GPCR	HCAR3	P49019	CHEMBL4421	Family A G protein-coupled receptor	0.0717871625222	1 / 0
DNA-(apurinic or apyrimidinic site) lyase	APEX1	P27695	CHEMBL5619	Enzyme	0.0717871625222	13 / 0
Lysine-specific demethylase 4B	KDM4B	O94953	CHEMBL3313832	Eraser	0.0717871625222	4 / 0
Lysine-specific demethylase 5B	KDM5B	Q9UGL1	CHEMBL3774295	Eraser	0.0717871625222	5 / 0
Cytidine deaminase	CDA	P32320	CHEMBL4502	Enzyme	0.0717871625222	25 / 0
Aldose reductase (by homology)	AKR1B1	P15121	CHEMBL1900	Enzyme	0.0717871625222	53 / 0
DNA excision repair protein ERCC-5	ERCC5	P28715	CHEMBL4736	Other nuclear protein	0.0717871625222	13 / 0
Estrogen receptor beta	ESR2	Q92731	CHEMBL242	Nuclear receptor	0.0717871625222	5 / 3
Dual-specificity tyrosine-phosphorylation regulated kinase 1A	DYRK1A	Q13627	CHEMBL2292	Kinase	0.0	14 / 0
Cyclin-dependent kinase 9	CDK9	P50750	CHEMBL3116	Kinase	0.0	8 / 0

Target	Common name	Uniprot ID	ChEMBL ID	Target Class	Probability*	Known actives (3D/2D)
Flap endonuclease 1	FEN1	P39748	CHEMBL5027	Enzyme	0.0	13 / 0
Dual specificty protein kinase CLK1 (by homology)	CLK1	P49759	CHEMBL4224	Kinase	0.0	13 / 0
CDK9/cyclin T1	CDK9 CCNT1	P50750 O60563	CHEMBL2111389	Other cytosolic protein	0.0	1 / 0
Casein kinase II alpha	CSNK2A1	P68400	CHEMBL3629	Kinase	0.0	8 / 0
C-C chemokine receptor type 2	CCR2	P41597	CHEMBL4015	Family A G protein-coupled receptor	0.0	3 / 0
Kynurenine 3-monooxygenase	KMO	O15229	CHEMBL2145	Oxidoreductase	0.0	36 / 0
Farnesyl diphosphate synthase	FDPS	P14324	CHEMBL1782	Transferase	0.0	2 / 0
Endothelin-converting enzyme 1	ECE1	P42892	CHEMBL4791	Protease	0.0	2 / 0
Alkaline phosphatase, tissue-nonspecific isozyme	ALPL	P05186	CHEMBL5979	Enzyme	0.0	6 / 0
Aldehyde reductase (by homology)	AKR1A1	P14550	CHEMBL2246	Enzyme	0.0	1 / 0
Thymidine kinase, cytosolic	TK1	P04183	CHEMBL2883	Transferase	0.0	10 / 0
DNA ligase 1	LIG1	P18858	CHEMBL5694	Enzyme	0.0	1 / 0
Peptidyl-glycine alpha-amidating monooxygenase	PAM	P19021	CHEMBL2544	Enzyme	0.0	1 / 0
Fructose-1,6-bisphosphatase	FBP1	P09467	CHEMBL3975	Enzyme	0.0	5 / 0
Neutral cholesterol ester hydrolase 1	NCEH1	Q6PIU2	CHEMBL5048	Enzyme	0.0	0 / 1
Angiotensin-converting enzyme (by homology)	ACE	P12821	CHEMBL1808	Protease	0.0	24 / 0
Poly [ADP-ribose] polymerase 14	PARP14	Q460N5	CHEMBL2176777	Enzyme	0.0	1 / 0
Neurotensin receptor 3	SORT1	Q99523	CHEMBL3091	Membrane receptor	0.0	10 / 0
Thymidylate synthase	TYMS	P04818	CHEMBL1952	Transferase	0.0	2 / 3
Tyrosyl-DNA phosphodiesterase 2	TDP2	O95551	CHEMBL2169736	Enzyme	0.0	1 / 0
Liver glycogen phosphorylase	PYGL	P06737	CHEMBL2568	Enzyme	0.0	19 / 0
Dual specificity tyrosine-phosphorylation-regulated kinase 1B	DYRK1B	Q9Y463	CHEMBL5543	Kinase	0.0	9 / 0
Dual specificity protein kinase CLK2 (by homology)	CLK2	P49760	CHEMBL4225	Kinase	0.0	9 / 0
Dual specificity protein kinase CLK4 (by homology)	CLK4	Q9HAZ1	CHEMBL4203	Kinase	0.0	10 / 0

Target	Common name	Uniprot ID	ChEMBL ID	Target Class	Probability*	Known actives (3D/2D)
Glutamate carboxypeptidase II	FOLH1	Q04609	CHEMBL1892	Protease	0.0	27 / 8
Beta-glucocerebrosidase	GBA	P04062	CHEMBL2179	Enzyme	0.0	9 / 0
Adenosine kinase	ADK	P55263	CHEMBL3589	Enzyme	0.0	12 / 0
Dihydroorotase	CAD	P27708	CHEMBL3093	Enzyme	0.0	2 / 0
Trypsin I	PRSS1	P07477	CHEMBL209	Protease	0.0	0 / 16
Acrosin	ACR	P10323	CHEMBL2738	Protease	0.0	0 / 16
Intercellular adhesion molecule (ICAM-1), Integrin alpha-L/ beta-2	ITGAL ICAM1 ITGB2	P20701 P05362 P05107	CHEMBL2096661	Membrane receptor	0.0	10 / 0
Tyrosyl-DNA phosphodiesterase 1	TDP1	Q9NUW8	CHEMBL1075138	Enzyme	0.0	1 / 0
Neprilysin (by homology)	MME	P08473	CHEMBL1944	Protease	0.0	11 / 0
Muscarinic acetylcholine receptor M1	CHRM1	P11229	CHEMBL216	Family A G protein-coupled receptor	0.0	1 / 0
Poly [ADP-ribose] polymerase 10	PARP10	Q53GL7	CHEMBL2429708	Enzyme	0.0	3 / 0
Aminopeptidase B	RNPEP	Q9H4A4	CHEMBL2432	Protease	0.0	2 / 0
Histone deacetylase 1	HDAC1	Q13547	CHEMBL325	Eraser	0.0	1 / 0
Hydroxyacid oxidase 2 (by homology)	HAO2	Q9NYQ3	CHEMBL2169732	Enzyme	0.0	7 / 0
Lysine-specific demethylase 6B	KDM6B	O15054	CHEMBL1938211	Eraser	0.0	8 / 0
Bifunctional protein NCOAT	OGA	O60502	CHEMBL5921	Enzyme	0.0	12 / 0
Glutamate [NMDA] receptor PROTEIN	GRIN1	Q05586	CHEMBL2015	Ligand-gated ion channel	0.0	1 / 0
Methionine aminopeptidase 2	METAP2	P50579	CHEMBL3922	Protease	0.0	2 / 0
Beta-hexosaminidase subunit alpha	HEXA	P06865	CHEMBL1250415	Enzyme	0.0	1 / 0
Beta-N-acetyl-D-hexosaminidase-A/B	HEXB	P07686	CHEMBL5877	Enzyme	0.0	1 / 0
Neuronal acetylcholine receptor; alpha4/ beta2	CHRNA4 CHRNB2	P43681 P17787	CHEMBL1907589	Ligand-gated ion channel	0.0	3 / 0
Casein kinase II alpha (prime)	CSNK2A2	P19784	CHEMBL4070	Kinase	0.0	3 / 0
Induced myeloid leukemia cell differentiation protein Mcl-1	MCL1	Q07820	CHEMBL4361	Other cytosolic protein	0.0	0 / 9
Palmitoleoyl-protein carboxylesterase NOTUM	NOTUM	Q6P988	CHEMBL3714531	Hydrolase	0.0	16 / 0
Poly [ADP-ribose] polymerase 15	PARP15	Q460N3	CHEMBL2176778	Enzyme	0.0	2 / 0

Target	Common name	Uniprot ID	ChEMBL ID	Target Class	Probability*	Known actives (3D/2D)
Alpha-L-fucosidase I	FUCA1	P04066	CHEMBL4176	Enzyme	0.0	10 / 0
Dual-specificity tyrosine-phosphorylation regulated kinase 2	DYRK2	Q92630	CHEMBL4376	Kinase	0.0	7 / 0
Solute carrier family 22 member 6	SLC22A6	Q4U2R8	CHEMBL1641347	Electrochemical transporter	0.0	1 / 0
Adenosine deaminase (by homology)	ADA	P00813	CHEMBL1910	Hydrolase	0.0	7 / 0
Matrix metalloproteinase 13	MMP13	P45452	CHEMBL280	Protease	0.0	2 / 0
Matrix metalloproteinase 12	MMP12	P39900	CHEMBL4393	Protease	0.0	2 / 0
Matrix metalloproteinase 8	MMP8	P22894	CHEMBL4588	Protease	0.0	2 / 0
Leukotriene A4 hydrolase	LTA4H	P09960	CHEMBL4618	Protease	0.0	2 / 0
Calpain 2	CAPN2	P17655	CHEMBL2382	Protease	0.0	2 / 0
Carboxypeptidase B2 isoform A	CPB2	Q96IY4	CHEMBL3419	Protease	0.0	6 / 0
Calpain 1	CAPN1	P07384	CHEMBL3891	Protease	0.0	2 / 0
Carboxypeptidase N, catalytic subunit	CPN1	P15169	CHEMBL4713	Protease	0.0	1 / 0
Transient receptor potential cation channel subfamily M member 8 (by homology)	TRPM8	Q7Z2W7	CHEMBL1075319	Voltage-gated ion channel	0.0	0 / 2
Lysine-specific demethylase 2A	KDM2A	Q9Y2K7	CHEMBL1938210	Eraser	0.0	6 / 0
Peptidyl-prolyl cis-trans isomerase NIMA-interacting 1	PIN1	Q13526	CHEMBL2288	Enzyme	0.0	1 / 0
Prolyl 4-hydroxylase subunit alpha-1	P4HA1	P13674	CHEMBL1250350	Enzyme	0.0	2 / 0
Tyrosine-protein kinase FYN	FYN	P06241	CHEMBL1841	Kinase	0.0	1 / 0
Tyrosine-protein kinase LCK	LCK	P06239	CHEMBL258	Kinase	0.0	2 / 0
G-protein coupled receptor kinase 2	GRK2	P25098	CHEMBL4079	Kinase	0.0	1 / 0
Lysine-specific demethylase 3A	KDM3A	Q9Y4C1	CHEMBL1938209	Eraser	0.0	3 / 0
Serine/threonine-protein kinase PIM1	PIM1	P11309	CHEMBL2147	Kinase	0.0	3 / 0
T1R1/ T1R3_UNCURATED	TAS1R3 TAS1R1	Q7RTX0 Q7RTX1	CHEMBL3832641	Family C G protein-coupled receptor	0.0	1 / 0
Hypoxia-inducible factor prolyl 4-hydroxylase	P4HTM	Q9NXG6	CHEMBL3047	Enzyme	0.0	6 / 0
Carboxypeptidase A1	CPA1	P15085	CHEMBL2088	Protease	0.0	8 / 0

Target	Common name	Uniprot ID	ChEMBL ID	Target Class	Probability*	Known actives (3D/2D)
Mast cell carboxypeptidase A	CPA3	P15088	CHEMBL2645	Protease	0.0	2 / 0
Dual specificity mitogen-activated protein kinase kinase 1	MAP2K1	Q02750	CHEMBL3587	Kinase	0.0	4 / 0
