# Supplementary material for: Identification of the Molecular Mechanisms of Peimine in the Treatment of Cough Using Computational Target Fishing
Source: Molecules. 2020 Mar 2;25(5):1105. doi: 10.3390/molecules25051105 (PMC7179178; doi:10.3390/molecules25051105)
Supplement: Supplementary file 1 [file molecules-25-01105-s001.zip › SwissTargetPrediction results of Chlorpheniramine.pdf]

SwissTargetPrediction

Target	Common name	Uniprot ID	ChEMBL ID	Target Class	Probability*	Known actives (3D/2D)
Histamine H1 receptor	HRH1	P35367	CHEMBL231	Family A G protein-coupled receptor	1.0	217 / 10
HERG	KCNH2	Q12809	CHEMBL240	Voltage-gated ion channel	1.0	551 / 27
Muscarinic acetylcholine receptor M4	CHRM4	P08173	CHEMBL1821	Family A G protein-coupled receptor	1.0	142 / 7
Alpha-2a adrenergic receptor	ADRA2A	P08913	CHEMBL1867	Family A G protein-coupled receptor	1.0	50 / 2
Histamine H2 receptor	HRH2	P25021	CHEMBL1941	Family A G protein-coupled receptor	1.0	21 / 3
Alpha-2b adrenergic receptor	ADRA2B	P18089	CHEMBL1942	Family A G protein-coupled receptor	1.0	32 / 3
Muscarinic acetylcholine receptor M5	CHRM5	P08912	CHEMBL2035	Family A G protein-coupled receptor	1.0	142 / 7
Muscarinic acetylcholine receptor M2	CHRM2	P08172	CHEMBL211	Family A G protein-coupled receptor	1.0	360 / 7
Muscarinic acetylcholine receptor M1	CHRM1	P11229	CHEMBL216	Family A G protein-coupled receptor	1.0	505 / 7
Norepinephrine transporter	SLC6A2	P23975	CHEMBL222	Electrochemical transporter	1.0	1282 / 83
Serotonin 2a (5-HT2a) receptor	HTR2A	P28223	CHEMBL224	Family A G protein-coupled receptor	1.0	976 / 12
Serotonin 2c (5-HT2c) receptor	HTR2C	P28335	CHEMBL225	Family A G protein-coupled receptor	1.0	509 / 11
Serotonin transporter	SLC6A4	P31645	CHEMBL228	Electrochemical transporter	1.0	2533 / 106
Dopamine transporter	SLC6A3	Q01959	CHEMBL238	Electrochemical transporter	1.0	2053 / 94
Muscarinic acetylcholine receptor M3	CHRM3	P20309	CHEMBL245	Family A G protein-coupled receptor	1.0	288 / 6
Sigma opioid receptor	SIGMAR1	Q99720	CHEMBL287	Membrane receptor	1.0	909 / 7
Histamine H3 receptor	HRH3	Q9Y5N1	CHEMBL264	Family A G protein-coupled receptor	0.112041901328	544 / 17
Focal adhesion kinase 1	PTK2	Q05397	CHEMBL2695	Kinase	0.112041901328	3 / 0
Mu opioid receptor	OPRM1	P35372	CHEMBL233	Family A G protein-coupled receptor	0.112041901328	880 / 65
Neuronal	CHRNA4	P43681	CHEMBL1882	Ligand-gated ion	0.112041901328	49 / 0

Target	Common name	Uniprot ID	ChEMBL ID	Target Class	Probability*	Known actives (3D/2D)
acetylcholine receptor protein alpha-4 subunit				channel		
Kappa Opioid receptor	OPRK1	P41145	CHEMBL237	Family A G protein-coupled receptor	0.112041901328	667 / 16
Neuronal acetylcholine receptor subunit alpha-3	CHRNA3	P32297	CHEMBL3068	Ligand-gated ion channel	0.112041901328	9 / 0
Progesterone receptor	PGR	P06401	CHEMBL208	Nuclear receptor	0.112041901328	14 / 0
Urotensin II receptor	UTS2R	Q9UKP6	CHEMBL3764	Family A G protein-coupled receptor	0.112041901328	81 / 0
Rho-associated protein kinase	ROCK2 ROCK1	O75116 Q13464	CHEMBL2111459	Kinase	0.112041901328	57 / 0
Neuronal acetylcholine receptor protein alpha-7 subunit	CHRNA7	P36544	CHEMBL2492	Ligand-gated ion channel	0.112041901328	192 / 0
Huntingtin	HTT	P42858	CHEMBL5514	Unclassified protein	0.112041901328	6 / 0
Neuronal acetylcholine receptor; alpha3/ beta4	CHRNA3 CHRNB4	P32297 P30926	CHEMBL1907594	Ligand-gated ion channel	0.112041901328	114 / 0
Lethal(3)malignant brain tumor-like protein 3	L3MBTL3	Q96JM7	CHEMBL1287623	Reader	0.112041901328	11 / 0
Nuclear receptor subfamily 1 group D member 1	NR1D1	P20393	CHEMBL1961783	Nuclear receptor	0.112041901328	6 / 0
Tyrosine-protein kinase JAK1	JAK1	P23458	CHEMBL2835	Kinase	0.112041901328	93 / 0
Dipeptidyl peptidase II	DPP7	Q9UHL4	CHEMBL3976	Protease	0.112041901328	165 / 0
Dipeptidyl peptidase VIII	DPP8	Q6V1X1	CHEMBL4657	Protease	0.112041901328	181 / 0
Dipeptidyl peptidase IX	DPP9	Q86TI2	CHEMBL4793	Protease	0.112041901328	129 / 0
Serotonin 4 (5-HT4) receptor	HTR4	Q13639	CHEMBL1875	Family A G protein-coupled receptor	0.112041901328	41 / 0
Tyrosine-protein kinase JAK3	JAK3	P52333	CHEMBL2148	Kinase	0.112041901328	38 / 0
Tyrosine-protein kinase JAK2	JAK2	O60674	CHEMBL2971	Kinase	0.112041901328	74 / 0
Tyrosine-protein kinase TYK2	TYK2	P29597	CHEMBL3553	Kinase	0.112041901328	19 / 0
Nociceptin receptor	OPRL1	P41146	CHEMBL2014	Family A G protein-coupled receptor	0.112041901328	349 / 70
Heat shock protein	TRAP1	Q12931	CHEMBL1075132	Other cytosolic	0.112041901328	3 / 0

Target	Common name	Uniprot ID	ChEMBL ID	Target Class	Probability*	Known actives (3D/2D)
75 kDa, mitochondrial				protein		
Endoplasmin	HSP90B1	P14625	CHEMBL1075323	Other membrane protein	0.112041901328	8 / 0
Histone deacetylase 2	HDAC2	Q92769	CHEMBL1937	Eraser	0.112041901328	1 / 0
Epidermal growth factor receptor erbB1	EGFR	P00533	CHEMBL203	Kinase	0.112041901328	159 / 0
Histone deacetylase 1	HDAC1	Q13547	CHEMBL325	Eraser	0.112041901328	39 / 0
Dopamine D2 receptor	DRD2	P14416	CHEMBL217	Family A G protein-coupled receptor	0.112041901328	2008 / 24
Dipeptidyl peptidase I	CTSC	P53634	CHEMBL2252	Protease	0.112041901328	63 / 0
Calcium-activated potassium channel subunit alpha-1	KCNMA1	Q12791	CHEMBL4304	Voltage-gated ion channel	0.112041901328	1 / 0
Stem cell growth factor receptor	KIT	P10721	CHEMBL1936	Kinase	0.112041901328	16 / 0
Alpha-1d adrenergic receptor	ADRA1D	P25100	CHEMBL223	Family A G protein-coupled receptor	0.112041901328	45 / 1
Alpha-1a adrenergic receptor	ADRA1A	P35348	CHEMBL229	Family A G protein-coupled receptor	0.112041901328	140 / 2
Alpha-1b adrenergic receptor	ADRA1B	P35368	CHEMBL232	Family A G protein-coupled receptor	0.112041901328	92 / 1
Peroxisome proliferator-activated receptor gamma	PPARG	P37231	CHEMBL235	Nuclear receptor	0.112041901328	1 / 0
Interleukin-1 receptor-associated kinase 4	IRAK4	Q9NWZ3	CHEMBL3778	Kinase	0.112041901328	47 / 0
Sodium channel protein type IV alpha subunit	SCN4A	P35499	CHEMBL2072	Voltage-gated ion channel	0.112041901328	13 / 0
Neuronal acetylcholine receptor; alpha3/ beta2	CHRNA3 CHRNB2	P32297 P17787	CHEMBL2109234	Ligand-gated ion channel	0.112041901328	11 / 0
Cytochrome P450 2D6	CYP2D6	P10635	CHEMBL289	Cytochrome P450	0.112041901328	15 / 0
Nicotinic acetylcholine receptor alpha4/ beta2/alpha5	CHRNA5 CHRNB2 CHRNA4	P30532 P17787 P43681	CHEMBL3038461	Ligand-gated ion channel	0.112041901328	1 / 0
Intercellular adhesion molecule (ICAM-1), Integrin alpha-L/ beta-2	ITGAL ICAM1 ITGB2	P20701 P05362 P05107	CHEMBL2096661	Membrane receptor	0.112041901328	2 / 0
CaM kinase II	CAMK2D	Q13557	CHEMBL2801	Kinase	0.112041901328	36 / 0
Rho-associated protein kinase 1	ROCK1	Q13464	CHEMBL3231	Kinase	0.112041901328	63 / 0

Target	Common name	Uniprot ID	ChEMBL ID	Target Class	Probability*	Known actives (3D/2D)
Fibroblast activation protein alpha	FAP	Q12884	CHEMBL4683	Protease	0.112041901328	42 / 0
Dopamine D4 receptor	DRD4	P21917	CHEMBL219	Family A G protein-coupled receptor	0.112041901328	502 / 19
Tyrosine-protein kinase ABL	ABL1	P00519	CHEMBL1862	Kinase	0.112041901328	4 / 0
Beta-3 adrenergic receptor	ADRB3	P13945	CHEMBL246	Family A G protein-coupled receptor	0.112041901328	4 / 0
Ribosomal protein S6 kinase alpha 5	RPS6KA5	O75582	CHEMBL4237	Kinase	0.112041901328	4 / 0
Ribosomal protein S6 kinase 1	RPS6KB1	P23443	CHEMBL4501	Kinase	0.112041901328	16 / 0
Serine/threonine-protein kinase Aurora-A	AURKA	O14965	CHEMBL4722	Kinase	0.112041901328	30 / 0
Serine/threonine-protein kinase D2	PRKD2	Q9BZL6	CHEMBL4900	Kinase	0.112041901328	1 / 0
MBT domain-containing protein 1	MBTD1	Q05BQ5	CHEMBL1287625	Reader	0.112041901328	2 / 0
Inhibitor of nuclear factor kappa B kinase beta subunit	IKBKB	O14920	CHEMBL1991	Kinase	0.112041901328	51 / 0
Myeloperoxidase	MPO	P05164	CHEMBL2439	Enzyme	0.112041901328	21 / 0
Inhibitor of NF-kappa-B kinase (IKK)	CHUK	O15111	CHEMBL3476	Kinase	0.112041901328	28 / 0
Acetylcholine receptor; alpha1/ beta1/delta/gamma	CHRNB1 CHRNA1 CHRNG CHRND	P11230 P02708 P07510 Q07001	CHEMBL1907588	Ligand-gated ion channel	0.112041901328	12 / 0
Serotonin 5a (5-HT5a) receptor	HTR5A	P47898	CHEMBL3426	Family A G protein-coupled receptor	0.112041901328	43 / 0
Rho GDP-dissociation inhibitor 1	ARHGDIA	P52565	CHEMBL3638327	Unclassified protein	0.112041901328	3 / 0
Lethal(3)malignant brain tumor-like protein 1	L3MBTL1	Q9Y468	CHEMBL1287622	Reader	0.112041901328	3 / 0
Myosin light chain kinase, smooth muscle	MYLK	Q15746	CHEMBL2428	Kinase	0.112041901328	6 / 0
Protein kinase C delta	PRKCD	Q05655	CHEMBL2996	Kinase	0.112041901328	98 / 0
Protein kinase C theta	PRKCQ	Q04759	CHEMBL3920	Kinase	0.112041901328	137 / 0
Leucine-rich repeat serine/threonine-protein kinase 2	LRRK2	Q5S007	CHEMBL1075104	Kinase	0.112041901328	5 / 0
Serotonin 1f (5-HT1f) receptor	HTR1F	P30939	CHEMBL1805	Family A G protein-coupled receptor	0.112041901328	73 / 0

Target	Common name	Uniprot ID	ChEMBL ID	Target Class	Probability*	Known actives (3D/2D)
TRAF2- and NCK-interacting kinase	TNIK	Q9UKE5	CHEMBL4527	Kinase	0.112041901328	1 / 0
Serine/threonine-protein kinase haspin	HASPIN	Q8TF76	CHEMBL1075163	Kinase	0.112041901328	12 / 0
Somatostatin receptor 3	SSTR3	P32745	CHEMBL2028	Family A G protein-coupled receptor	0.112041901328	41 / 0
Multidrug resistance-associated protein 1	ABCC1	P33527	CHEMBL3004	Primary active transporter	0.112041901328	31 / 0
ATP-binding cassette sub-family G member 2	ABCG2	Q9UNQ0	CHEMBL5393	Primary active transporter	0.112041901328	3 / 0
P2X purinoceptor 7	P2RX7	Q99572	CHEMBL4805	Ligand-gated ion channel	0.112041901328	5 / 0
Hormone sensitive lipase	LIPE	Q05469	CHEMBL3590	Enzyme	0.112041901328	1 / 0
c-Jun N-terminal kinase 1	MAPK8	P45983	CHEMBL2276	Kinase	0.112041901328	16 / 0
c-Jun N-terminal kinase 2	MAPK9	P45984	CHEMBL4179	Kinase	0.112041901328	5 / 0
Ubiquitin carboxyl-terminal hydrolase 14	USP14	P54578	CHEMBL1293295	Enzyme	0.112041901328	1 / 0
Serine/threonine-protein kinase PIM1	PIM1	P11309	CHEMBL2147	Kinase	0.112041901328	240 / 0
Tyrosine-protein kinase FYN	FYN	P06241	CHEMBL1841	Kinase	0.112041901328	5 / 0
Prolyl endopeptidase	PREP	P48147	CHEMBL3202	Protease	0.112041901328	12 / 0
Cytochrome P450 1A2	CYP1A2	P05177	CHEMBL3356	Cytochrome P450	0.112041901328	3 / 0
Amine oxidase, copper containing	AOC3	Q16853	CHEMBL3437	Enzyme	0.112041901328	5 / 0
Metabotropic glutamate receptor 3	GRM3	Q14832	CHEMBL2888	Family C G protein-coupled receptor	0.112041901328	2 / 0
Squalene synthetase (by homology)	FDFT1	P37268	CHEMBL3338	Enzyme	0.112041901328	14 / 0
Vesicular acetylcholine transporter	SLC18A3	Q16572	CHEMBL4767	Electrochemical transporter	0.112041901328	8 / 0
Kinesin-1 heavy chain/ Tyrosine-protein kinase receptor RET	RET	P07949	CHEMBL2041	Kinase	0.112041901328	16 / 0
