# Supplementary material for: Identification of the Molecular Mechanisms of Peimine in the Treatment of Cough Using Computational Target Fishing
Source: Molecules. 2020 Mar 2;25(5):1105. doi: 10.3390/molecules25051105 (PMC7179178; doi:10.3390/molecules25051105)
Supplement: Supplementary file 1 [file molecules-25-01105-s001.zip › SwissTargetPrediction results of Dextromethorphan.pdf]

SwissTargetPrediction

Target	Common name	Uniprot ID	ChEMBL ID	Target Class	Probability*	Known actives (3D/2D)
Serotonin transporter	SLC6A4	P31645	CHEMBL228	Electrochemical transporter	1.0	1472 / 862
Sigma opioid receptor	SIGMAR1	Q99720	CHEMBL287	Membrane receptor	1.0	621 / 717
Dopamine D2 receptor (by homology)	DRD2	P14416	CHEMBL217	Family A G protein-coupled receptor	0.673980204067	1102 / 1097
Neuronal acetylcholine receptor; alpha4/beta2	CHRNA4 CHRNB2	P43681 P17787	CHEMBL1907589	Ligand-gated ion channel	0.395446541133	173 / 38
Neuronal acetylcholine receptor; alpha3/beta4	CHRNA3 CHRNB4	P32297 P30926	CHEMBL1907594	Ligand-gated ion channel	0.395446541133	96 / 19
Neuronal acetylcholine receptor protein alpha-7 subunit	CHRNA7	P36544	CHEMBL2492	Ligand-gated ion channel	0.395446541133	132 / 8
Mu opioid receptor	OPRM1	P35372	CHEMBL233	Family A G protein-coupled receptor	0.370888463379	674 / 1730
Dopamine D4 receptor	DRD4	P21917	CHEMBL219	Family A G protein-coupled receptor	0.354464973753	222 / 127
Dopamine D3 receptor	DRD3	P35462	CHEMBL234	Family A G protein-coupled receptor	0.329868485421	411 / 543
Kappa Opioid receptor	OPRK1	P41145	CHEMBL237	Family A G protein-coupled receptor	0.329868485421	483 / 1035
Serotonin 1a (5-HT1a) receptor	HTR1A	P08908	CHEMBL214	Family A G protein-coupled receptor	0.321690506669	447 / 249
Glutamate NMDA receptor; GRIN1/ GRIN2A	GRIN2A GRIN1	Q12879 Q05586	CHEMBL1907604	Ligand-gated ion channel	0.288928252457	5 / 10
Delta opioid receptor	OPRD1	P41143	CHEMBL236	Family A G protein-coupled receptor	0.288928252457	479 / 1407
Norepinephrine transporter	SLC6A2	P23975	CHEMBL222	Electrochemical transporter	0.239574695827	930 / 423
Dopamine transporter	SLC6A3	Q01959	CHEMBL238	Electrochemical transporter	0.239574695827	1291 / 774
Dopamine D1 receptor	DRD1	P21728	CHEMBL2056	Family A G protein-coupled receptor	0.231637184894	176 / 184
Alpha-1d adrenergic receptor	ADRA1D	P25100	CHEMBL223	Family A G protein-coupled receptor	0.157929217109	13 / 35
Alpha-1a adrenergic receptor	ADRA1A	P35348	CHEMBL229	Family A G protein-coupled receptor	0.157929217109	22 / 82
Alpha-1b adrenergic receptor	ADRA1B	P35368	CHEMBL232	Family A G protein-coupled receptor	0.149732593856	15 / 55
Dopamine D5 receptor	DRD5	P21918	CHEMBL1850	Family A G	0.141522086267	75 / 67

Target	Common name	Uniprot ID	ChEMBL ID	Target Class	Probability*	Known actives (3D/2D)
				protein-coupled receptor		
Serotonin 5a (5-HT5a) receptor	HTR5A	P47898	CHEMBL3426	Family A G protein-coupled receptor	0.133337961498	35 / 23
Muscarinic acetylcholine receptor M2	CHRM2	P08172	CHEMBL211	Family A G protein-coupled receptor	0.133337961498	152 / 57
11-beta-hydroxysteroid dehydrogenase 1	HSD11B1	P28845	CHEMBL4235	Enzyme	0.133337961498	26 / 286
Anti-estrogen binding site (AEBS)	EBP	Q15125	CHEMBL4931	Enzyme	0.125142648574	3 / 13
Synaptic vesicular amine transporter (by homology)	SLC18A2	Q05940	CHEMBL1893	Electrochemical transporter	0.116965063224	16 / 162
Muscarinic acetylcholine receptor M1	CHRM1	P11229	CHEMBL216	Family A G protein-coupled receptor	0.116965063224	254 / 47
Serotonin 1b (5-HT1b) receptor (by homology)	HTR1B	P28222	CHEMBL1898	Family A G protein-coupled receptor	0.116965063224	169 / 52
Steroid 5-alpha-reductase 1	SRD5A1	P18405	CHEMBL1787	Oxidoreductase	0.108770969359	0 / 33
Glutamate [NMDA] receptor subunit epsilon 2	GRIN2B	Q13224	CHEMBL1904	Ligand-gated ion channel	0.108770969359	7 / 18
Serotonin 2c (5-HT2c) receptor	HTR2C	P28335	CHEMBL225	Family A G protein-coupled receptor	0.108770969359	244 / 133
Serotonin 2a (5-HT2a) receptor	HTR2A	P28223	CHEMBL224	Family A G protein-coupled receptor	0.100578902067	492 / 387
Alpha-2a adrenergic receptor	ADRA2A	P08913	CHEMBL1867	Family A G protein-coupled receptor	0.100578902067	16 / 18
Adrenergic receptor alpha-2	ADRA2C	P18825	CHEMBL1916	Family A G protein-coupled receptor	0.100578902067	30 / 23
Serotonin 2b (5-HT2b) receptor	HTR2B	P41595	CHEMBL1833	Family A G protein-coupled receptor	0.100578902067	126 / 86
Histamine H2 receptor	HRH2	P25021	CHEMBL1941	Family A G protein-coupled receptor	0.100578902067	17 / 21
Serotonin 7 (5-HT7) receptor	HTR7	P34969	CHEMBL3155	Family A G protein-coupled receptor	0.100578902067	172 / 35
Alpha-2b adrenergic receptor	ADRA2B	P18089	CHEMBL1942	Family A G protein-coupled receptor	0.100578902067	16 / 15
Serotonin 1d (5-HT1d) receptor	HTR1D	P28221	CHEMBL1983	Family A G protein-coupled receptor	0.100578902067	127 / 48
Histamine H3 receptor	HRH3	Q9Y5N1	CHEMBL264	Family A G	0.100578902067	248 /

Target	Common name	Uniprot ID	ChEMBL ID	Target Class	Probability*	Known actives (3D/2D)
				protein-coupled receptor		296
DNA polymerase alpha subunit	POLA1	P09884	CHEMBL1828	Transferase	0.100578902067	1 / 13
Dipeptidyl peptidase IV	DPP4	P27487	CHEMBL284	Protease	0.100578902067	452 / 103
Lysine-specific histone demethylase 1	KDM1A	O60341	CHEMBL6136	Eraser	0.100578902067	70 / 55
Adenosine A3 receptor	ADORA3	P0DMS8	CHEMBL256	Family A G protein-coupled receptor	0.100578902067	3 / 5
Neuropeptide Y receptor type 5	NPY5R	Q15761	CHEMBL4561	Family A G protein-coupled receptor	0.100578902067	2 / 40
P-glycoprotein 1	ABCB1	P08183	CHEMBL4302	Primary active transporter	0.100578902067	5 / 52
Voltage-gated L-type calcium channel alpha-1C subunit	CACNA1C	Q13936	CHEMBL1940	Voltage-gated ion channel	0.100578902067	0 / 3
HERG	KCNH2	Q12809	CHEMBL240	Voltage-gated ion channel	0.100578902067	239 / 118
Acetylcholinesterase	ACHE	P22303	CHEMBL220	Hydrolase	0.100578902067	75 / 210
Melatonin receptor 1A	MTNR1A	P48039	CHEMBL1945	Family A G protein-coupled receptor	0.100578902067	2 / 167
Melatonin receptor 1B	MTNR1B	P49286	CHEMBL1946	Family A G protein-coupled receptor	0.100578902067	4 / 155
Putative hydrolase RBBP9	RBBP9	O75884	CHEMBL1075121	Enzyme	0.100578902067	0 / 1
Histamine H1 receptor	HRH1	P35367	CHEMBL231	Family A G protein-coupled receptor	0.100578902067	131 / 45
Small conductance calcium-activated potassium channel protein 1	KCNN1	Q92952	CHEMBL2369	Voltage-gated ion channel	0.100578902067	0 / 1
Small conductance calcium-activated potassium channel protein 3	KCNN3	Q9UGI6	CHEMBL3381	Voltage-gated ion channel	0.100578902067	0 / 1
Small conductance calcium-activated potassium channel protein 2	KCNN2	Q9H2S1	CHEMBL4469	Voltage-gated ion channel	0.100578902067	0 / 1
Toll-like receptor 4 (by homology)	TLR4	O00206	CHEMBL5255	Toll-like and Il-1 receptors	0.100578902067	0 / 11
Solute carrier family 22 member 1	SLC22A1	O15245	CHEMBL5685	Electrochemical transporter	0.100578902067	0 / 1
PH domain leucine-rich repeat-containing protein phosphatase 2	PHLPP2	Q6ZVD8	CHEMBL1275209	Enzyme	0.100578902067	1 / 0
Nociceptin receptor	OPRL1	P41146	CHEMBL2014	Family A G protein-coupled	0.100578902067	187 / 93

Target	Common name	Uniprot ID	ChEMBL ID	Target Class	Probability*	Known actives (3D/2D)
				receptor		
Sodium channel protein type IV alpha subunit	SCN4A	P35499	CHEMBL2072	Voltage-gated ion channel	0.100578902067	13 / 0
LXR-alpha	NR1H3	Q13133	CHEMBL2808	Nuclear receptor	0.100578902067	0 / 25
LXR-beta	NR1H2	P55055	CHEMBL4093	Nuclear receptor	0.100578902067	0 / 24
Rho-associated protein kinase 2	ROCK2	O75116	CHEMBL2973	Kinase	0.100578902067	95 / 0
Rho-associated protein kinase	ROCK2 ROCK1	O75116 Q13464	CHEMBL2111459	Kinase	0.100578902067	50 / 0
Rho-associated protein kinase 1	ROCK1	Q13464	CHEMBL3231	Kinase	0.100578902067	29 / 0
Serotonin 1e (5-HT1e) receptor	HTR1E	P28566	CHEMBL2182	Family A G protein-coupled receptor	0.100578902067	10 / 0
Neuronal acetylcholine receptor; alpha3/ alpha6/beta2/beta3	CHRNB3 CHRNA6 CHRNB2 CHRNA3	Q05901 Q15825 P17787 P32297	CHEMBL2109233	Ligand-gated ion channel	0.100578902067	7 / 0
Muscarinic acetylcholine receptor M5	CHRM5	P08912	CHEMBL2035	Family A G protein-coupled receptor	0.100578902067	64 / 28
Muscarinic acetylcholine receptor M3	CHRM3	P20309	CHEMBL245	Family A G protein-coupled receptor	0.100578902067	106 / 50
Tyrosine-protein kinase JAK3	JAK3	P52333	CHEMBL2148	Kinase	0.100578902067	14 / 0
Tyrosine-protein kinase JAK1	JAK1	P23458	CHEMBL2835	Kinase	0.100578902067	43 / 0
Tyrosine-protein kinase JAK2	JAK2	O60674	CHEMBL2971	Kinase	0.100578902067	33 / 0
Tyrosine-protein kinase TYK2	TYK2	P29597	CHEMBL3553	Kinase	0.100578902067	11 / 0
Carbonic anhydrase II	CA2	P00918	CHEMBL205	Lyase	0.100578902067	22 / 24
Carbonic anhydrase I	CA1	P00915	CHEMBL261	Lyase	0.100578902067	13 / 17
Quinone reductase 2	NQO2	P16083	CHEMBL3959	Enzyme	0.100578902067	5 / 31
Tyrosine-protein kinase LCK	LCK	P06239	CHEMBL258	Kinase	0.100578902067	1 / 1
Glutamate NMDA receptor; GRIN1/ GRIN2B	GRIN1 GRIN2B	Q05586 Q13224	CHEMBL1907603	Ligand-gated ion channel	0.100578902067	3 / 22
Arachidonate 5-lipoxygenase	ALOX5	P09917	CHEMBL215	Oxidoreductase	0.100578902067	0 / 16
Rho GDP-dissociation inhibitor 1	ARHGDIA	P52565	CHEMBL3638327	Unclassified protein	0.100578902067	3 / 0
Serotonin 3a (5-HT3a) receptor	HTR3A	P46098	CHEMBL1899	Ligand-gated ion channel	0.100578902067	73 / 0
Butyrylcholinesterase	BCHE	P06276	CHEMBL1914	Hydrolase	0.100578902067	24 / 34
Nitric-oxide synthase, brain	NOS1	P29475	CHEMBL3568	Enzyme	0.100578902067	73 / 0
Histamine H4 receptor	HRH4	Q9H3N8	CHEMBL3759	Family A G protein-coupled	0.100578902067	88 / 0

Target	Common name	Uniprot ID	ChEMBL ID	Target Class	Probability*	Known actives (3D/2D)
				receptor		
Tyrosine-protein kinase FYN	FYN	P06241	CHEMBL1841	Kinase	0.100578902067	0 / 2
Carbonic anhydrase VII	CA7	P43166	CHEMBL2326	Lyase	0.100578902067	0 / 1
Carbonic anhydrase III	CA3	P07451	CHEMBL2885	Lyase	0.100578902067	0 / 1
Carbonic anhydrase VI	CA6	P23280	CHEMBL3025	Lyase	0.100578902067	0 / 8
Carbonic anhydrase IV	CA4	P22748	CHEMBL3729	Lyase	0.100578902067	0 / 8
Carbonic anhydrase XIII	CA13	Q8N1Q1	CHEMBL3912	Lyase	0.100578902067	0 / 1
Carbonic anhydrase VB	CA5B	Q9Y2D0	CHEMBL3969	Lyase	0.100578902067	0 / 1
Carbonic anhydrase VA	CA5A	P35218	CHEMBL4789	Lyase	0.100578902067	0 / 1
Calcium-activated potassium channel subunit alpha-1	KCNMA1	Q12791	CHEMBL4304	Voltage-gated ion channel	0.100578902067	1 / 0
Cytochrome P450 24A1 (by homology)	CYP24A1	Q07973	CHEMBL4521	Enzyme	0.100578902067	0 / 7
Indoleamine 2,3-dioxygenase	IDO1	P14902	CHEMBL4685	Enzyme	0.100578902067	1 / 0
Protein tyrosine phosphatase receptor type C-associated protein	PTPRCAP	Q14761	CHEMBL4806	Enzyme	0.100578902067	1 / 0
Serotonin 6 (5-HT6) receptor	HTR6	P50406	CHEMBL3371	Family A G protein-coupled receptor	0.0	233 / 27
Epoxide hydratase	EPHX2	P34913	CHEMBL2409	Protease	0.0	6 / 33
Neurokinin 1 receptor	TACR1	P25103	CHEMBL249	Family A G protein-coupled receptor	0.0	68 / 146
Neuronal acetylcholine receptor protein alpha-4 subunit	CHRNA4	P43681	CHEMBL1882	Ligand-gated ion channel	0.0	27 / 0
