# Supplementary material for: Identification of the Molecular Mechanisms of Peimine in the Treatment of Cough Using Computational Target Fishing
Source: Molecules. 2020 Mar 2;25(5):1105. doi: 10.3390/molecules25051105 (PMC7179178; doi:10.3390/molecules25051105)
Supplement: Supplementary file 1 [file molecules-25-01105-s001.zip › SwissTargetPrediction results of Homatropine.pdf]

SwissTargetPrediction

Target	Common name	Uniprot ID	ChEMBL ID	Target Class	Probability*	Known actives (3D/2D)
Muscarinic acetylcholine receptor M4	CHRM4	P08173	CHEMBL1821	Family A G protein-coupled receptor	0.912946448399	53 / 59
Muscarinic acetylcholine receptor M5	CHRM5	P08912	CHEMBL2035	Family A G protein-coupled receptor	0.912946448399	47 / 48
Muscarinic acetylcholine receptor M2	CHRM2	P08172	CHEMBL211	Family A G protein-coupled receptor	0.912946448399	256 / 223
Muscarinic acetylcholine receptor M1	CHRM1	P11229	CHEMBL216	Family A G protein-coupled receptor	0.912946448399	263 / 319
Muscarinic acetylcholine receptor M3	CHRM3	P20309	CHEMBL245	Family A G protein-coupled receptor	0.912946448399	212 / 213
Acetylcholinesterase (by homology)	ACHE	P22303	CHEMBL220	Hydrolase	0.198833760448	83 / 3
Alpha-1d adrenergic receptor	ADRA1D	P25100	CHEMBL223	Family A G protein-coupled receptor	0.198833760448	94 / 7
Serotonin 2c (5-HT2c) receptor	HTR2C	P28335	CHEMBL225	Family A G protein-coupled receptor	0.198833760448	179 / 7
Sigma opioid receptor	SIGMAR1	Q99720	CHEMBL287	Membrane receptor	0.108770969359	353 / 125
Dopamine transporter (by homology)	SLC6A3	Q01959	CHEMBL238	Electrochemical transporter	0.100578902067	818 / 1229
Norepinephrine transporter	SLC6A2	P23975	CHEMBL222	Electrochemical transporter	0.100578902067	562 / 214
Serotonin transporter	SLC6A4	P31645	CHEMBL228	Electrochemical transporter	0.100578902067	995 / 754
Neuronal acetylcholine receptor protein alpha-7 subunit	CHRNA7	P36544	CHEMBL2492	Ligand-gated ion channel	0.100578902067	94 / 12
Polyadenylate-binding protein 1	PABPC1	P11940	CHEMBL1293286	Unclassified protein	0.100578902067	1 / 5
Serotonin 2a (5-HT2a) receptor	HTR2A	P28223	CHEMBL224	Family A G protein-coupled receptor	0.100578902067	315 / 21
Serotonin 2b (5-HT2b) receptor	HTR2B	P41595	CHEMBL1833	Family A G protein-coupled receptor	0.100578902067	105 / 6
Poly [ADP-ribose] polymerase-1	PARP1	P09874	CHEMBL3105	Enzyme	0.100578902067	121 / 0
Monoamine oxidase A	MAOA	P21397	CHEMBL1951	Oxidoreductase	0.100578902067	14 / 0
Serotonin 1f (5-HT1f) receptor	HTR1F	P30939	CHEMBL1805	Family A G protein-coupled receptor	0.100578902067	20 / 0
Alpha-1a adrenergic receptor (by homology)	ADRA1A	P35348	CHEMBL229	Family A G protein-coupled receptor	0.100578902067	160 / 10
Troponin, cardiac muscle	TNNC1 TNNT2	P63316 P45379	CHEMBL2095202	Unclassified protein	0.100578902067	3 / 0

Target	Common name	Uniprot ID	ChEMBL ID	Target Class	Probability*	Known actives (3D/2D)
	TNNI3	P19429				
Transient receptor potential cation channel subfamily V member 3	TRPV3	Q8NET8	CHEMBL5522	Voltage-gated ion channel	0.100578902067	88 / 0
Delta opioid receptor	OPRD1	P41143	CHEMBL236	Family A G protein-coupled receptor	0.100578902067	788 / 24
Serine/threonine-protein kinase Aurora-B	AURKB	Q96GD4	CHEMBL2185	Kinase	0.100578902067	69 / 0
Ribosomal protein S6 kinase 1	RPS6KB1	P23443	CHEMBL4501	Kinase	0.100578902067	92 / 0
Serine/threonine-protein kinase Aurora-A	AURKA	O14965	CHEMBL4722	Kinase	0.100578902067	159 / 0
Dopamine D3 receptor	DRD3	P35462	CHEMBL234	Family A G protein-coupled receptor	0.100578902067	237 / 47
Serotonin 6 (5-HT6) receptor	HTR6	P50406	CHEMBL3371	Family A G protein-coupled receptor	0.100578902067	262 / 3
Glycogen synthase kinase-3 beta	GSK3B	P49841	CHEMBL262	Kinase	0.100578902067	132 / 0
Glycogen synthase kinase-3 alpha	GSK3A	P49840	CHEMBL2850	Kinase	0.100578902067	22 / 0
Dipeptidyl peptidase II	DPP7	Q9UHL4	CHEMBL3976	Protease	0.100578902067	59 / 0
Fibroblast activation protein alpha	FAP	Q12884	CHEMBL4683	Protease	0.100578902067	9 / 0
Dopamine D2 receptor	DRD2	P14416	CHEMBL217	Family A G protein-coupled receptor	0.100578902067	545 / 66
Histamine H4 receptor	HRH4	Q9H3N8	CHEMBL3759	Family A G protein-coupled receptor	0.100578902067	133 / 0
Dipeptidyl peptidase I	CTSC	P53634	CHEMBL2252	Protease	0.100578902067	10 / 0
Serine/threonine-protein kinase ILK-1	ILK	Q13418	CHEMBL5247	Kinase	0.100578902067	70 / 0
Glutamate [NMDA] receptor subunit epsilon 2	GRIN2B	Q13224	CHEMBL1904	Ligand-gated ion channel	0.100578902067	7 / 0
MAP kinase p38 alpha (by homology)	MAPK14	Q16539	CHEMBL260	Kinase	0.100578902067	114 / 0
MAP kinase p38 beta	MAPK11	Q15759	CHEMBL3961	Kinase	0.100578902067	21 / 0
c-Jun N-terminal kinase 2	MAPK9	P45984	CHEMBL4179	Kinase	0.100578902067	29 / 0
Phenylethanolamine N-methyltransferase	PNMT	P11086	CHEMBL4617	Enzyme	0.100578902067	10 / 0
Alpha-2a adrenergic receptor	ADRA2A	P08913	CHEMBL1867	Family A G protein-coupled receptor	0.100578902067	68 / 5
Alpha-2b adrenergic receptor	ADRA2B	P18089	CHEMBL1942	Family A G protein-coupled receptor	0.100578902067	39 / 4
Xaa-Pro aminopeptidase 1	XPNPEP1	Q9NQW7	CHEMBL3782	Protease	0.100578902067	2 / 0
Leucine aminopeptidase	LAP3	P28838	CHEMBL3965	Protease	0.100578902067	8 / 0

Target	Common name	Uniprot ID	ChEMBL ID	Target Class	Probability*	Known actives (3D/2D)
Xaa-Pro dipeptidase	PEPD	P12955	CHEMBL4185	Protease	0.100578902067	4 / 0
Xaa-Pro aminopeptidase 2	XPNPEP2	O43895	CHEMBL4610	Protease	0.100578902067	4 / 0
Trace amine-associated receptor 1 (by homology)	TAAR1	Q96RJ0	CHEMBL5857	Family A G protein-coupled receptor	0.100578902067	200 / 0
Histamine H1 receptor	HRH1	P35367	CHEMBL231	Family A G protein-coupled receptor	0.100578902067	122 / 15
Plasminogen	PLG	P00747	CHEMBL1801	Protease	0.100578902067	1 / 0
Metastin receptor	KISS1R	Q969F8	CHEMBL5413	Family A G protein-coupled receptor	0.100578902067	4 / 0
Bromodomain-containing protein 4	BRD4	O60885	CHEMBL1163125	Reader	0.100578902067	52 / 0
Tyrosine-protein kinase JAK1	JAK1	P23458	CHEMBL2835	Kinase	0.100578902067	200 / 0
Melanin-concentrating hormone receptor 1	MCHR1	Q99705	CHEMBL344	Family A G protein-coupled receptor	0.100578902067	51 / 0
MAP kinase ERK2	MAPK1	P28482	CHEMBL4040	Kinase	0.100578902067	144 / 0
Methionine aminopeptidase 2	METAP2	P50579	CHEMBL3922	Protease	0.100578902067	71 / 0
DNA topoisomerase II alpha	TOP2A	P11388	CHEMBL1806	Isomerase	0.100578902067	5 / 0
Glycine transporter 1	SLC6A9	P48067	CHEMBL2337	Electrochemical transporter	0.100578902067	64 / 0
Androgen Receptor (by homology)	AR	P10275	CHEMBL1871	Nuclear receptor	0.100578902067	141 / 0
Phosphodiesterase 9A	PDE9A	O76083	CHEMBL3535	Phosphodiesterase	0.100578902067	25 / 0
Phosphodiesterase 1C	PDE1C	Q14123	CHEMBL4619	Phosphodiesterase	0.100578902067	13 / 0
S-methyl-5-thioadenosine phosphorylase	MTAP	Q13126	CHEMBL4941	Enzyme	0.100578902067	37 / 0
Histamine H3 receptor	HRH3	Q9Y5N1	CHEMBL264	Family A G protein-coupled receptor	0.100578902067	207 / 26
Cyclin-dependent kinase 9	CDK9	P50750	CHEMBL3116	Kinase	0.100578902067	46 / 0
Adenosine A3 receptor	ADORA3	P0DMS8	CHEMBL256	Family A G protein-coupled receptor	0.100578902067	162 / 3
Lysine-specific histone demethylase 1	KDM1A	O60341	CHEMBL6136	Eraser	0.100578902067	8 / 0
Mu opioid receptor	OPRM1	P35372	CHEMBL233	Family A G protein-coupled receptor	0.100578902067	902 / 79
Dual specificity mitogen-activated protein kinase kinase 1	MAP2K1	Q02750	CHEMBL3587	Kinase	0.100578902067	150 / 0
Ghrelin receptor	GHSR	Q92847	CHEMBL4616	Family A G protein-coupled receptor	0.100578902067	24 / 0
Interleukin-1	IRAK4	Q9NWZ3	CHEMBL3778	Kinase	0.100578902067	44 / 0

Target	Common name	Uniprot ID	ChEMBL ID	Target Class	Probability*	Known actives (3D/2D)
receptor-associated kinase 4						
Cytochrome P450 51	CYP51A1	Q16850	CHEMBL3849	Cytochrome P450	0.100578902067	12 / 0
Protein arginine N-methyltransferase 6	PRMT6	Q96LA8	CHEMBL1275221	Writer	0.100578902067	3 / 0
Neuronal acetylcholine receptor; alpha3/alpha6/beta2/beta3	CHRNB3 CHRNA6 CHRNB2 CHRNA3	Q05901 Q15825 P17787 P32297	CHEMBL2109233	Ligand-gated ion channel	0.100578902067	4 / 0
Protein arginine N-methyltransferase 8	PRMT8	Q9NR22	CHEMBL3108648	Writer	0.100578902067	3 / 0
Cytochrome P450 1A2	CYP1A2	P05177	CHEMBL3356	Cytochrome P450	0.100578902067	3 / 0
Dipeptidyl peptidase VIII	DPP8	Q6V1X1	CHEMBL4657	Protease	0.100578902067	60 / 0
Protein-arginine N-methyltransferase 1	PRMT1	Q99873	CHEMBL5524	Writer	0.100578902067	3 / 0
Leucine-rich repeat serine/threonine-protein kinase 2	LRRK2	Q5S007	CHEMBL1075104	Kinase	0.100578902067	92 / 0
Rho-associated protein kinase	ROCK2 ROCK1	O75116 Q13464	CHEMBL2111459	Kinase	0.100578902067	9 / 0
Aspartyl aminopeptidase	DNPEP	Q9ULA0	CHEMBL2761	Protease	0.100578902067	2 / 0
Serine/threonine-protein kinase AKT	AKT1	P31749	CHEMBL4282	Kinase	0.100578902067	117 / 0
Phosphodiesterase 10A	PDE10A	Q9Y233	CHEMBL4409	Phosphodiesterase	0.100578902067	313 / 0
Nitric oxide synthase, inducible	NOS2	P35228	CHEMBL4481	Enzyme	0.100578902067	43 / 0
Kappa Opioid receptor	OPRK1	P41145	CHEMBL237	Family A G protein-coupled receptor	0.100578902067	595 / 31
Phosphodiesterase 7A	PDE7A	Q13946	CHEMBL3012	Phosphodiesterase	0.100578902067	15 / 0
Indoleamine 2,3-dioxygenase	IDO1	P14902	CHEMBL4685	Enzyme	0.100578902067	7 / 0
Dipeptidyl peptidase IX	DPP9	Q86TI2	CHEMBL4793	Protease	0.100578902067	35 / 0
Urotensin II receptor	UTS2R	Q9UKP6	CHEMBL3764	Family A G protein-coupled receptor	0.100578902067	25 / 0
Bromodomain adjacent to zinc finger domain protein 2B	BAZ2B	Q9UIF8	CHEMBL1741220	Reader	0.100578902067	10 / 0
Bromodomain adjacent to zinc finger domain protein 2A	BAZ2A	Q9UIF9	CHEMBL3108642	Reader	0.100578902067	9 / 0
Cyclin-dependent kinase 2/cyclin A	CDK2 CCNA1 CCNA2	P24941 P78396 P20248	CHEMBL2094128	Other cytosolic protein	0.100578902067	89 / 0
Carbonic anhydrase VII	CA7	P43166	CHEMBL2326	Lyase	0.100578902067	16 / 0

Target	Common name	Uniprot ID	ChEMBL ID	Target Class	Probability*	Known actives (3D/2D)
Carbonic anhydrase I	CA1	P00915	CHEMBL261	Lyase	0.100578902067	85 / 0
Carbonic anhydrase XII	CA12	O43570	CHEMBL3242	Lyase	0.100578902067	42 / 0
Carbonic anhydrase IX	CA9	Q16790	CHEMBL3594	Lyase	0.100578902067	64 / 0
Carbonic anhydrase XIII	CA13	Q8N1Q1	CHEMBL3912	Lyase	0.100578902067	18 / 0
Serine/threonine-protein kinase Chk1	CHEK1	O14757	CHEMBL4630	Kinase	0.100578902067	136 / 0
HERG	KCNH2	Q12809	CHEMBL240	Voltage-gated ion channel	0.100578902067	336 / 35
Tyrosine-protein kinase receptor FLT3	FLT3	P36888	CHEMBL1974	Kinase	0.100578902067	76 / 0
Phosphodiesterase 11A	PDE11A	Q9HCR9	CHEMBL2717	Phosphodiesterase	0.100578902067	8 / 0
