# Supplementary material for: Identification of the Molecular Mechanisms of Peimine in the Treatment of Cough Using Computational Target Fishing
Source: Molecules. 2020 Mar 2;25(5):1105. doi: 10.3390/molecules25051105 (PMC7179178; doi:10.3390/molecules25051105)
Supplement: Supplementary file 1 [file molecules-25-01105-s001.zip › SwissTargetPrediction results of montelukast.pdf]

SwissTargetPrediction

Target	Common name	Uniprot ID	ChEMBL ID	Target Class	Probability*	Known actives (3D/2D)
Uracil nucleotide/ cysteinyl leukotriene receptor	GPR17	Q13304	CHEMBL1075162	Family A G protein-coupled receptor	1.0	1 / 1
Cysteinyl leukotriene receptor 1	CYSLTR1	Q9Y271	CHEMBL1798	Family A G protein-coupled receptor	1.0	33 / 37
Serotonin 2b (5-HT2b) receptor	HTR2B	P41595	CHEMBL1833	Family A G protein-coupled receptor	1.0	1 / 1
Thromboxane-A synthase	TBXAS1	P24557	CHEMBL1835	Cytochrome P450	1.0	201 / 1
Tyrosine-protein kinase FYN	FYN	P06241	CHEMBL1841	Kinase	1.0	3 / 1
Alpha-2a adrenergic receptor	ADRA2A	P08913	CHEMBL1867	Family A G protein-coupled receptor	1.0	2 / 1
Adrenergic receptor alpha-2	ADRA2C	P18825	CHEMBL1916	Family A G protein-coupled receptor	1.0	2 / 1
Epidermal growth factor receptor erbB1	EGFR	P00533	CHEMBL203	Kinase	1.0	34 / 1
Adrenergic receptor beta	ADRB2	P07550	CHEMBL210	Family A G protein-coupled receptor	1.0	4 / 1
Muscarinic acetylcholine receptor M1	CHRM1	P11229	CHEMBL216	Family A G protein-coupled receptor	1.0	5 / 1
Norepinephrine transporter	SLC6A2	P23975	CHEMBL222	Electrochemical transporter	1.0	1 / 1
Neurokinin 2 receptor	TACR2	P21452	CHEMBL2327	Family A G protein-coupled receptor	1.0	22 / 1
Dopamine D3 receptor	DRD3	P35462	CHEMBL234	Family A G protein-coupled receptor	1.0	7 / 1
Delta opioid receptor	OPRD1	P41143	CHEMBL236	Family A G protein-coupled receptor	1.0	265 / 1
Dopamine transporter	SLC6A3	Q01959	CHEMBL238	Electrochemical transporter	1.0	16 / 1
Muscarinic acetylcholine receptor M3	CHRM3	P20309	CHEMBL245	Family A G protein-coupled receptor	1.0	2 / 1
Beta-3 adrenergic receptor	ADRB3	P13945	CHEMBL246	Family A G protein-coupled receptor	1.0	5 / 1
Adenosine A3 receptor	ADORA3	P0DMS8	CHEMBL256	Family A G protein-coupled receptor	1.0	17 / 1
MAP kinase p38 alpha	MAPK14	Q16539	CHEMBL260	Kinase	1.0	85 / 1

Target	Common name	Uniprot ID	ChEMBL ID	Target Class	Probability*	Known actives (3D/2D)
Sphingosine 1-phosphate receptor Edg-5	S1PR2	O95136	CHEMBL2955	Family A G protein-coupled receptor	0.0743922719035	21 / 0
Protein farnesyltransferase	FNTA FNTB	P49354 P49356	CHEMBL2094108	Enzyme	0.0743922719035	192 / 0
Cannabinoid receptor 1	CNR1	P21554	CHEMBL218	Family A G protein-coupled receptor	0.0743922719035	41 / 0
Cannabinoid receptor 2	CNR2	P34972	CHEMBL253	Family A G protein-coupled receptor	0.0743922719035	41 / 0
Pepsinogen C (by homology)	PGC	P20142	CHEMBL2136	Protease	0.0743922719035	1 / 0
Type-1 angiotensin II receptor (by homology)	AGTR1	P30556	CHEMBL227	Family A G protein-coupled receptor	0.0743922719035	228 / 0
Renin	REN	P00797	CHEMBL286	Protease	0.0743922719035	106 / 0
Prostanoid EP4 receptor (by homology)	PTGER4	P35408	CHEMBL1836	Family A G protein-coupled receptor	0.0743922719035	214 / 0
Prostanoid EP2 receptor (by homology)	PTGER2	P43116	CHEMBL1881	Family A G protein-coupled receptor	0.0743922719035	153 / 0
FK506-binding protein 1A	FKBP1A	P62942	CHEMBL1902	Isomerase	0.0743922719035	79 / 0
Peptidyl-prolyl cis-trans isomerase FKBP5	FKBP5	Q13451	CHEMBL2052031	Enzyme	0.0743922719035	17 / 0
Cholecystokinin B receptor	CCKBR	P32239	CHEMBL298	Family A G protein-coupled receptor	0.0743922719035	515 / 0
FK506 binding protein 4	FKBP4	Q02790	CHEMBL4050	Enzyme	0.0743922719035	10 / 0
Cathepsin D	CTSD	P07339	CHEMBL2581	Protease	0.0743922719035	17 / 0
Inosine-5'-monophosphate dehydrogenase 1	IMPDH1	P20839	CHEMBL1822	Oxidoreductase	0.0743922719035	11 / 0
Inosine-5'-monophosphate dehydrogenase 2	IMPDH2	P12268	CHEMBL2002	Oxidoreductase	0.0743922719035	13 / 0
Endothelin receptor ET-A (by homology)	EDNRA	P25101	CHEMBL252	Family A G protein-coupled receptor	0.0743922719035	465 / 0
Leukocyte elastase	ELANE	P08246	CHEMBL248	Protease	0.0743922719035	38 / 0
Mu opioid receptor	OPRM1	P35372	CHEMBL233	Family A G protein-coupled receptor	0.0743922719035	216 / 0
Kappa Opioid receptor	OPRK1	P41145	CHEMBL237	Family A G protein-coupled receptor	0.0743922719035	65 / 0
Ileal bile acid transporter	SLC10A2	Q12908	CHEMBL2778	Electrochemical transporter	0.0743922719035	33 / 0
Glucagon receptor	GCGR	P47871	CHEMBL1985	Family B G	0.0743922719035	159 / 0

Target	Common name	Uniprot ID	ChEMBL ID	Target Class	Probability*	Known actives (3D/2D)
				protein-coupled receptor		
Integrin alpha-4	ITGA4	P13612	CHEMBL278	Membrane receptor	0.0743922719035	144 / 0
G protein-coupled receptor 44	PTGDR2	Q9Y5Y4	CHEMBL5071	Family A G protein-coupled receptor	0.0743922719035	417 / 0
Epoxide hydratase	EPHX2	P34913	CHEMBL2409	Protease	0.0743922719035	48 / 0
Vitamin D receptor	VDR	P11473	CHEMBL1977	Nuclear receptor	0.0743922719035	25 / 0
Arachidonate 5-lipoxygenase	ALOX5	P09917	CHEMBL215	Oxidoreductase	0.0743922719035	122 / 0
Phospholipase A2 group 1VB	PLA2G4B	P0C869	CHEMBL4136	Enzyme	0.0743922719035	10 / 0
Metabotropic glutamate receptor 2 (by homology)	GRM2	Q14416	CHEMBL5137	Family C G protein-coupled receptor	0.0743922719035	30 / 0
Prostaglandin E synthase	PTGES	O14684	CHEMBL5658	Enzyme	0.0743922719035	104 / 0
Squalene synthetase (by homology)	FDFT1	P37268	CHEMBL3338	Enzyme	0.0743922719035	79 / 0
Endothelin receptor ET-B	EDNRB	P24530	CHEMBL1785	Family A G protein-coupled receptor	0.0743922719035	171 / 0
Integrin alpha-4/ beta-7	ITGB7 ITGA4	P26010 P13612	CHEMBL2095184	Membrane receptor	0.0743922719035	265 / 0
Caspase-3	CASP3	P42574	CHEMBL2334	Protease	0.0743922719035	161 / 0
Caspase-1 (by homology)	CASP1	P29466	CHEMBL4801	Protease	0.0743922719035	247 / 0
Integrin alpha-4/ beta-1	ITGB1 ITGA4	P05556 P13612	CHEMBL1907599	Membrane receptor	0.0743922719035	618 / 0
Thymidylate synthase (by homology)	TYMS	P04818	CHEMBL1952	Transferase	0.0743922719035	58 / 0
Thromboxane A2 receptor	TBXA2R	P21731	CHEMBL2069	Family A G protein-coupled receptor	0.0743922719035	158 / 0
Prostanoid EP1 receptor (by homology)	PTGER1	P34995	CHEMBL1811	Family A G protein-coupled receptor	0.0743922719035	65 / 0
Cholecystokinin A receptor (by homology)	CCKAR	P32238	CHEMBL1901	Family A G protein-coupled receptor	0.0743922719035	75 / 0
Peroxisome proliferator-activated receptor gamma	PPARG	P37231	CHEMBL235	Nuclear receptor	0.0743922719035	872 / 0
Histamine H1 receptor	HRH1	P35367	CHEMBL231	Family A G protein-coupled receptor	0.0743922719035	83 / 0
Neurokinin 1 receptor	TACR1	P25103	CHEMBL249	Family A G protein-coupled receptor	0.0743922719035	14 / 0
Methionine aminopeptidase 2	METAP2	P50579	CHEMBL3922	Protease	0.0743922719035	88 / 0

Target	Common name	Uniprot ID	ChEMBL ID	Target Class	Probability*	Known actives (3D/2D)
Angiotensin II receptor	AGTR2	P50052	CHEMBL4607	Family A G protein-coupled receptor	0.0743922719035	62 / 0
Inhibitor of nuclear factor kappa B kinase beta subunit	IKBKB	O14920	CHEMBL1991	Kinase	0.0743922719035	17 / 0
Thioredoxin	TXN	P10599	CHEMBL2010624	Unclassified protein	0.0743922719035	1 / 0
Thioredoxin, mitochondrial	TXN2	Q99757	CHEMBL2189153	Unclassified protein	0.0743922719035	1 / 0
Induced myeloid leukemia cell differentiation protein Mcl-1	MCL1	Q07820	CHEMBL4361	Other cytosolic protein	0.0743922719035	48 / 0
Calcium sensing receptor	CASR	P41180	CHEMBL1878	Family C G protein-coupled receptor	0.0743922719035	30 / 0
Purinergic receptor P2Y12	P2RY12	Q9H244	CHEMBL2001	Family A G protein-coupled receptor	0.0743922719035	120 / 0
C-C chemokine receptor type 5	CCR5	P51681	CHEMBL274	Family A G protein-coupled receptor	0.0743922719035	126 / 0
Opioid growth factor receptor-like protein 1	OGFRL1	Q5TC84	CHEMBL3638334	Unclassified protein	0.0743922719035	30 / 0
Plectin	PLEC	Q15149	CHEMBL1293240	Unclassified protein	0.0743922719035	5 / 0
Integrin alpha-V/ beta-3	ITGAV ITGB3	P06756 P05106	CHEMBL1907598	Membrane receptor	0.0	511 / 0
Matrix metalloproteinase 3	MMP3	P08254	CHEMBL283	Protease	0.0	195 / 0
Matrix metalloproteinase 2	MMP2	P08253	CHEMBL333	Protease	0.0	297 / 0
Sphingosine 1-phosphate receptor Edg-1	S1PR1	P21453	CHEMBL4333	Family A G protein-coupled receptor	0.0	60 / 0
C-X-C chemokine receptor type 3	CXCR3	P49682	CHEMBL4441	Family A G protein-coupled receptor	0.0	137 / 0
Nuclear receptor ROR-gamma	RORC	P51449	CHEMBL1741186	Nuclear receptor	0.0	6 / 0
Cytosolic phospholipase A2	PLA2G4A	P47712	CHEMBL3816	Enzyme	0.0	82 / 0
Bombesin receptor subtype-3	BRS3	P32247	CHEMBL4080	Family A G protein-coupled receptor	0.0	7 / 0
Tyrosine-protein kinase LCK	LCK	P06239	CHEMBL258	Kinase	0.0	12 / 0
Caspase-7	CASP7	P55210	CHEMBL3468	Protease	0.0	79 / 0
Caspase-8	CASP8	Q14790	CHEMBL3776	Protease	0.0	75 / 0
Endothelin-converting enzyme 1	ECE1	P42892	CHEMBL4791	Protease	0.0	128 / 0

Target	Common name	Uniprot ID	ChEMBL ID	Target Class	Probability*	Known actives (3D/2D)
Integrin alpha-IIb/ beta-3	ITGA2B ITGB3	P08514 P05106	CHEMBL2093869	Membrane receptor	0.0	191 / 0
Integrin alpha-5/ beta-1	ITGB1 ITGA5	P05556 P08648	CHEMBL2095226	Membrane receptor	0.0	51 / 0
Integrin alpha-V/ beta-5	ITGB5 ITGAV	P18084 P06756	CHEMBL2096675	Membrane receptor	0.0	67 / 0
Serine/threonine-protein kinase Aurora-B	AURKB	Q96GD4	CHEMBL2185	Kinase	0.0	30 / 0
Cathepsin S	CTSS	P25774	CHEMBL2954	Protease	0.0	33 / 0
Ubiquitin-like domain-containing CTD phosphatase 1	UBLCP1	Q8WVY7	CHEMBL3317333	Enzyme	0.0	4 / 0
Serine/threonine-protein kinase Aurora-A	AURKA	O14965	CHEMBL4722	Kinase	0.0	36 / 0
Membrane-associated guanylate kinase-related 3	MAGI3	Q5TCQ9	CHEMBL5212	Enzyme	0.0	4 / 0
Cyclin-dependent kinase 5/CDK5 activator 1	CDK5R1 CDK5	Q15078 Q00535	CHEMBL1907600	Kinase	0.0	33 / 0
Neprilysin (by homology)	MME	P08473	CHEMBL1944	Protease	0.0	158 / 0
HERG	KCNH2	Q12809	CHEMBL240	Voltage-gated ion channel	0.0	34 / 0
Protein phosphatase 2A regulatory subunit B'	PTPA	Q15257	CHEMBL2505	Phosphatase	0.0	3 / 0
Platelet activating factor receptor	PTAFR	P25105	CHEMBL250	Family A G protein-coupled receptor	0.0	4 / 0
C-C chemokine receptor type 3	CCR3	P51677	CHEMBL3473	Family A G protein-coupled receptor	0.0	52 / 0
TRAIL receptor-1	TNFRSF10A	O00220	CHEMBL3551	Membrane receptor	0.0	1 / 0
