# Supplementary material for: Identification of the Molecular Mechanisms of Peimine in the Treatment of Cough Using Computational Target Fishing
Source: Molecules. 2020 Mar 2;25(5):1105. doi: 10.3390/molecules25051105 (PMC7179178; doi:10.3390/molecules25051105)
Supplement: Supplementary file 1 [file molecules-25-01105-s001.zip › SwissTargetPrediction Results of R048-8071.pdf]

SwissTargetPrediction

Target	Common name	Uniprot ID	ChEMBL ID	Target Class	Probability*	Known actives (3D/2D)
Lanosterol synthase	LSS	P48449	CHEMBL3593	Enzyme	1.0	77 / 54
Somatostatin receptor 5	SSTR5	P35346	CHEMBL1792	Family A G protein-coupled receptor	0.11573667475	87 / 0
Vasopressin V1a receptor	AVPR1A	P37288	CHEMBL1889	Family A G protein-coupled receptor	0.11573667475	171 / 0
Poly [ADP-ribose] polymerase-1	PARP1	P09874	CHEMBL3105	Enzyme	0.11573667475	647 / 0
Lysosomal Pro-X carboxypeptidase	PRCP	P42785	CHEMBL2335	Protease	0.11573667475	176 / 0
Melanocortin receptor 4	MC4R	P32245	CHEMBL259	Family A G protein-coupled receptor	0.11573667475	1072 / 0
Maternal embryonic leucine zipper kinase	MELK	Q14680	CHEMBL4578	Kinase	0.11573667475	243 / 0
Vascular endothelial growth factor receptor 2	KDR	P35968	CHEMBL279	Kinase	0.11573667475	621 / 0
Aurora kinase B/Inner centromere protein	INCENP AURKB	Q9NQS7 Q96GD4	CHEMBL3430907	Kinase	0.11573667475	10 / 0
Dual specificity protein kinase TTK	TTK	P33981	CHEMBL3983	Kinase	0.11573667475	46 / 0
Serotonin 1f (5-HT1f) receptor	HTR1F	P30939	CHEMBL1805	Family A G protein-coupled receptor	0.11573667475	90 / 0
Voltage-gated T-type calcium channel alpha-1H subunit	CACNA1H	O95180	CHEMBL1859	Voltage-gated ion channel	0.11573667475	31 / 0
Serotonin 1d (5-HT1d) receptor	HTR1D	P28221	CHEMBL1983	Family A G protein-coupled receptor	0.11573667475	558 / 0
Thrombin and coagulation factor X	F10	P00742	CHEMBL244	Protease	0.11573667475	588 / 0
Inhibitor of nuclear factor kappa B kinase epsilon subunit	IKBKE	Q14164	CHEMBL3529	Kinase	0.11573667475	18 / 0
Serine/threonine-protein kinase TBK1	TBK1	Q9UHD2	CHEMBL5408	Kinase	0.11573667475	27 / 0
Neuropeptide Y receptor type 1	NPY1R	P25929	CHEMBL4777	Family A G protein-coupled receptor	0.11573667475	170 / 0
Corticotropin releasing factor receptor 1	CRHR1	P34998	CHEMBL1800	Family B G protein-coupled receptor	0.11573667475	44 / 0
MAP kinase ERK2 (by	MAPK1	P28482	CHEMBL4040	Kinase	0.11573667475	180 / 0

Target	Common name	Uniprot ID	ChEMBL ID	Target Class	Probability*	Known actives (3D/2D)
homology)						
Acetylcholinesterase	ACHE	P22303	CHEMBL220	Hydrolase	0.11573667475	842 / 19
Serotonin 1b (5-HT1b) receptor	HTR1B	P28222	CHEMBL1898	Family A G protein-coupled receptor	0.11573667475	604 / 0
Serine/threonine-protein kinase mTOR	MTOR	P42345	CHEMBL2842	Kinase	0.11573667475	252 / 0
Protein kinase C delta	PRKCD	Q05655	CHEMBL2996	Kinase	0.11573667475	170 / 0
Protein kinase C theta	PRKCQ	Q04759	CHEMBL3920	Kinase	0.11573667475	277 / 0
Voltage-gated T-type calcium channel alpha-1I subunit	CACNA1I	Q9P0X4	CHEMBL5558	Voltage-gated ion channel	0.11573667475	18 / 0
Liver glycogen phosphorylase	PYGL	P06737	CHEMBL2568	Enzyme	0.11573667475	10 / 0
PI3-kinase p110-alpha subunit	PIK3CA	P42336	CHEMBL4005	Enzyme	0.11573667475	222 / 0
Tyrosine-protein kinase ZAP-70	ZAP70	P43403	CHEMBL2803	Kinase	0.11573667475	50 / 0
Tyrosine-protein kinase JAK2	JAK2	O60674	CHEMBL2971	Kinase	0.11573667475	292 / 0
Lysine-specific histone demethylase 1	KDM1A	O60341	CHEMBL6136	Eraser	0.11573667475	89 / 0
Phospholipase D1	PLD1	Q13393	CHEMBL2536	Hydrolase	0.11573667475	72 / 0
Phospholipase D2	PLD2	O14939	CHEMBL2734	Hydrolase	0.11573667475	61 / 0
Ghrelin receptor	GHSR	Q92847	CHEMBL4616	Family A G protein-coupled receptor	0.11573667475	314 / 0
Tyrosine-protein kinase receptor UFO	AXL	P30530	CHEMBL4895	Kinase	0.11573667475	62 / 0
Proto-oncogene tyrosine-protein kinase MER	MERTK	Q12866	CHEMBL5331	Kinase	0.11573667475	41 / 0
Serotonin 4 (5-HT4) receptor	HTR4	Q13639	CHEMBL1875	Family A G protein-coupled receptor	0.11573667475	107 / 0
Serotonin 3a (5-HT3a) receptor	HTR3A	P46098	CHEMBL1899	Ligand-gated ion channel	0.11573667475	209 / 0
Inhibitor of nuclear factor kappa B kinase beta subunit	IKBKB	O14920	CHEMBL1991	Kinase	0.11573667475	207 / 0
Inhibitor of NF-kappa-B kinase (IKK)	IKBKG IKBKB CHUK	Q9Y6K9 O14920 O15111	CHEMBL2111328	Kinase	0.11573667475	10 / 0
Serine/threonine-protein kinase Aurora-B	AURKB	Q96GD4	CHEMBL2185	Kinase	0.11573667475	184 / 0
Tyrosine-protein kinase LCK	LCK	P06239	CHEMBL258	Kinase	0.11573667475	176 / 0
Inhibitor of NF-kappa-B kinase (IKK)	CHUK	O15111	CHEMBL3476	Kinase	0.11573667475	71 / 0

Target	Common name	Uniprot ID	ChEMBL ID	Target Class	Probability*	Known actives (3D/2D)
Tyrosine-protein kinase BRK	PTK6	Q13882	CHEMBL4601	Kinase	0.11573667475	26 / 0
Cyclin-dependent kinase 2/cyclin A	CDK2 CCNA1 CCNA2	P24941 P78396 P20248	CHEMBL2094128	Other cytosolic protein	0.11573667475	154 / 0
MAP kinase-activated protein kinase 2	MAPKAPK2	P49137	CHEMBL2208	Kinase	0.11573667475	262 / 0
Rho-associated protein kinase 2	ROCK2	O75116	CHEMBL2973	Kinase	0.11573667475	543 / 0
C-C chemokine receptor type 3	CCR3	P51677	CHEMBL3473	Family A G protein-coupled receptor	0.11573667475	442 / 0
Receptor protein-tyrosine kinase erbB-2	ERBB2	P04626	CHEMBL1824	Kinase	0.11573667475	230 / 0
Protein kinase C alpha	PRKCA	P17252	CHEMBL299	Kinase	0.11573667475	101 / 0
Protein kinase C beta	PRKCB	P05771	CHEMBL3045	Kinase	0.11573667475	220 / 0
Protein kinase C epsilon	PRKCE	Q02156	CHEMBL3582	Kinase	0.11573667475	67 / 0
Macrophage colony stimulating factor receptor	CSF1R	P07333	CHEMBL1844	Kinase	0.11573667475	217 / 0
C-C chemokine receptor type 4	CCR4	P51679	CHEMBL2414	Family A G protein-coupled receptor	0.11573667475	86 / 0
Somatostatin receptor 2	SSTR2	P30874	CHEMBL1804	Family A G protein-coupled receptor	0.11573667475	160 / 0
Somatostatin receptor 4	SSTR4	P31391	CHEMBL1853	Family A G protein-coupled receptor	0.11573667475	49 / 0
Gonadotropin-releasing hormone receptor	GNRHR	P30968	CHEMBL1855	Family A G protein-coupled receptor	0.11573667475	395 / 0
Somatostatin receptor 1	SSTR1	P30872	CHEMBL1917	Family A G protein-coupled receptor	0.11573667475	57 / 0
Somatostatin receptor 3	SSTR3	P32745	CHEMBL2028	Family A G protein-coupled receptor	0.11573667475	122 / 0
MAP kinase p38 alpha	MAPK14	Q16539	CHEMBL260	Kinase	0.11573667475	321 / 0
Tyrosine-protein kinase receptor FLT3	FLT3	P36888	CHEMBL1974	Kinase	0.11573667475	165 / 0
Centromere-associated protein E	CENPE	Q02224	CHEMBL5870	Unclassified protein	0.11573667475	11 / 0
Vascular endothelial growth factor receptor 1	FLT1	P17948	CHEMBL1868	Kinase	0.11573667475	99 / 0
Rho-associated protein kinase	ROCK2 ROCK1	O75116 Q13464	CHEMBL2111459	Kinase	0.11573667475	57 / 0

Target	Common name	Uniprot ID	ChEMBL ID	Target Class	Probability*	Known actives (3D/2D)
Histamine N-methyltransferase	HNMT	P50135	CHEMBL2190	Enzyme	0.11573667475	7 / 0
Fibroblast growth factor receptor 1	FGFR1	P11362	CHEMBL3650	Kinase	0.11573667475	204 / 0
Hepatocyte growth factor receptor	MET	P08581	CHEMBL3717	Kinase	0.11573667475	339 / 0
Inhibitor of apoptosis protein 3	XIAP	P98170	CHEMBL4198	Other cytosolic protein	0.11573667475	443 / 0
Nitric-oxide synthase, brain	NOS1	P29475	CHEMBL3568	Enzyme	0.11573667475	264 / 0
Serine/threonine-protein kinase AKT	AKT1	P31749	CHEMBL4282	Kinase	0.11573667475	770 / 0
Nucleotide-binding oligomerization domain-containing protein 1	NOD1	Q9Y239	CHEMBL1293222	Unclassified protein	0.11573667475	9 / 0
Nucleotide-binding oligomerization domain-containing protein 2	NOD2	Q9HC29	CHEMBL1293266	Unclassified protein	0.11573667475	8 / 0
Platelet-derived growth factor receptor beta	PDGFRB	P09619	CHEMBL1913	Kinase	0.11573667475	131 / 0
Stem cell growth factor receptor	KIT	P10721	CHEMBL1936	Kinase	0.11573667475	126 / 0
Histamine H4 receptor	HRH4	Q9H3N8	CHEMBL3759	Family A G protein-coupled receptor	0.11573667475	282 / 0
Prostanoid EP1 receptor	PTGER1	P34995	CHEMBL1811	Family A G protein-coupled receptor	0.11573667475	21 / 0
c-Jun N-terminal kinase 1	MAPK8	P45983	CHEMBL2276	Kinase	0.11573667475	74 / 0
c-Jun N-terminal kinase 3	MAPK10	P53779	CHEMBL2637	Kinase	0.11573667475	61 / 0
PI3-kinase p110-delta subunit	PIK3CD	O00329	CHEMBL3130	Enzyme	0.11573667475	91 / 0
Membrane-bound transcription factor site-1 protease	MBTPS1	Q14703	CHEMBL5916	Protease	0.11573667475	18 / 0
Nociceptin receptor	OPRL1	P41146	CHEMBL2014	Family A G protein-coupled receptor	0.11573667475	533 / 0
TGF-beta receptor type I	TGFBR1	P36897	CHEMBL4439	Kinase	0.11573667475	74 / 0
Melanocortin receptor 5	MC5R	P33032	CHEMBL4608	Family A G protein-coupled receptor	0.11573667475	163 / 0
Melanocortin receptor 3	MC3R	P41968	CHEMBL4644	Family A G protein-coupled receptor	0.11573667475	189 / 0
Cell division protein	CDK8	P49336	CHEMBL5719	Kinase	0.11573667475	10 / 0

Target	Common name	Uniprot ID	ChEMBL ID	Target Class	Probability*	Known actives (3D/2D)
kinase 8						
Neurokinin 2 receptor	TACR2	P21452	CHEMBL2327	Family A G protein-coupled receptor	0.11573667475	215 / 0
Cyclin-dependent kinase 9	CDK9	P50750	CHEMBL3116	Kinase	0.11573667475	174 / 0
Melanocortin receptor 1	MC1R	Q01726	CHEMBL3795	Family A G protein-coupled receptor	0.11573667475	177 / 0
Tyrosine-protein kinase ABL	ABL1	P00519	CHEMBL1862	Kinase	0.11573667475	128 / 0
Cyclin-dependent kinase 4/cyclin D1	CCND1 CDK4	P24385 P11802	CHEMBL1907601	Kinase	0.11573667475	86 / 0
Thrombin	F2	P00734	CHEMBL204	Protease	0.11573667475	526 / 0
Ceramide glucosyltransferase	UGCG	Q16739	CHEMBL2063	Transferase	0.11573667475	10 / 0
Protein farnesyltransferase	FNTA FNTB	P49354 P49356	CHEMBL2094108	Enzyme	0.11573667475	183 / 0
Adrenergic receptor beta	ADRB2	P07550	CHEMBL210	Family A G protein-coupled receptor	0.11573667475	39 / 0
Beta-1 adrenergic receptor	ADRB1	P08588	CHEMBL213	Family A G protein-coupled receptor	0.11573667475	58 / 0
Serotonin 1e (5-HT1e) receptor	HTR1E	P28566	CHEMBL2182	Family A G protein-coupled receptor	0.11573667475	11 / 0
Serotonin 5a (5-HT5a) receptor	HTR5A	P47898	CHEMBL3426	Family A G protein-coupled receptor	0.11573667475	60 / 0
Urotensin II receptor	UTS2R	Q9UKP6	CHEMBL3764	Family A G protein-coupled receptor	0.11573667475	199 / 0
Nitric-oxide synthase, endothelial	NOS3	P29474	CHEMBL4803	Enzyme	0.11573667475	82 / 0
Dopamine D2 receptor (by homology)	DRD2	P14416	CHEMBL217	Family A G protein-coupled receptor	0.11573667475	4310 / 38
Sigma opioid receptor	SIGMAR1	Q99720	CHEMBL287	Membrane receptor	0.11573667475	1243 / 35
